# Alterations of Epigenetic Signatures in Hepatocyte Nuclear Factor 4α Deficient Mouse Liver Determined by Improved ChIP-qPCR and (h)MeDIP-qPCR Assays

**DOI:** 10.1371/journal.pone.0084925

**Published:** 2014-01-10

**Authors:** Qinghao Zhang, Xiaohong Lei, Hong Lu

**Affiliations:** Department of Pharmacology, SUNY Upstate Medical University, Syracuse, New York, United States of America; Univeristy of California Riverside, United States of America

## Abstract

Hepatocyte nuclear factor 4**α** (HNF4**α**) is a liver-enriched transcription factor essential for liver development and function. In hepatocytes, HNF4**α** regulates a large number of genes important for nutrient/xenobiotic metabolism and cell differentiation and proliferation. Currently, little is known about the epigenetic mechanism of gene regulation by HNF4**α**. In this study, the global and specific alterations at the selected gene loci of representative histone modifications and DNA methylations were investigated in *Hnf4a*-deficient female mouse livers using the improved MeDIP-, hMeDIP- and ChIP-qPCR assay. *Hnf4a* deficiency significantly increased hepatic total IPed DNA fragments for histone H3 lysine-4 dimethylation (H3K4me2), H3K4me3, H3K9me2, H3K27me3 and H3K4 acetylation, but not for H3K9me3, 5-methylcytosine,or 5-hydroxymethylcytosine. At specific gene loci, the relative enrichments of histone and DNA modifications were changed to different degree in *Hnf4a*-deficient mouse liver. Among the epigenetic signatures investigated, changes in H3K4me3 correlated the best with mRNA expression. Additionally, *Hnf4a*-deficient livers had increased mRNA expression of histone H1.2 and H3.3 as well as epigenetic modifiers *Dnmt1*, *Tet3*, *Setd7*, *Kmt2c*, *Ehmt2*, and *Ezh2*. In conclusion, the present study provides convenient improved (h)MeDIP- and ChIP-qPCR assays for epigenetic study. *Hnf4a* deficiency in young-adult mouse liver markedly alters histone methylation and acetylation, with fewer effects on DNA methylation and 5-hydroxymethylation. The underlying mechanism may be the induction of epigenetic enzymes responsible for the addition/removal of the epigenetic signatures, and/or the loss of HNF4**α** *per se* as a key coordinator for epigenetic modifiers.

## Introduction

HNF4α is a conserved member of the nuclear receptor superfamily of ligand-dependent transcription factors [1]. As a liver-enriched transcription factor, HNF4α is also expressed in the kidney, small intestine, colon, stomach, and pancreas in which the mutation of *HNF4A* gene causes maturity-onset diabetes of the young in humans (MODY) [2], [3]. HNF4α is essential in liver development and differentiation, lipid homeostasis, bile acid synthesis, as well as the expression of phase I, II, and III drug processing genes [4]–[6]. Aberrations in HNF4α functionality are linked to development of severe cirrhotic livers, alcoholic liver disease, tumor necrosis factor-α-induced hepatotoxicity, and hepatocellular carcinoma where HNF4α has antiproliferative effect and serves as a tumor suppressor [4], [5], [7]–[9]. The number of potential target genes of HNF4α has been estimated to be thousands in genome-wide analyses, and these genes encode proteins implicated in a wide variety of biological processes [4], [10], [11]. Additionally, HNF4α may interact with other nuclear receptors such as chicken ovalbumin upstream promoter-transcription factor, retinoid X receptor, peroxisome proliferator-activated receptor (PPAR), farnesoid X receptor, constitutive androstane receptor, glucocorticoid receptor, Vitamin D receptor, and small heterodimer partner to directly or indirectly regulate gene expression [1], [2], [12].

Chromatin is the complex of DNA and histone proteins, which provides the scaffold for the packaging of entire genome [13]. Modifications on DNA and histone proteins of chromatin are two main categories of epigenetic modifications that play crucial roles in the development and differentiation of various cell types, normal cellular processes, and diseases such as cancer [14], [15]. Dawson and Kouzarides discussed in a review that it is time to embrace the central role of epigenetics in cancer [13]. At present, there are at least four different DNA modifications and 16 classes of histone modifications reported [13]. Histone modifications include methylation, acetylation, ubiquitination, phosphorylation etc. In recent years, considerable progress in understanding histone methylation and acetylation has been achieved, and histone methylations including histone H3 lysine 4 (H3K4), H3K9, H3K27, H3K36, H3K79 and H4K20 have been extensively studied [15], [16]. Although the methylation of 5-carbon on cytosine residues (5 mC) was initially considered a relatively stable DNA modification, later studies indicate that the ten-eleven translocation (TET) family of proteins have the ability to convert 5 mC to 5-hydroxymethylcytosine (5 hmC) which can be further oxidized to 5-formylcytosine and 5-carboxylcytosine [13].

There are limited studies on the chromatin-related alterations by HNF4α, although previous study has suggested that HNF4**α** regulating gene expression may be mediated by its influence on epigenetic modifications [9]. HNF4 and HNF1α are considered to be involved in establishing the reorganization of chromatin within serpin gene cluster at 14q32.1 to control the activities of two cell-specific genes α1-antitrypsin and corticosteroid-binding globulin [17]. The coactivators, such as steroid receptor coactivator-1, glucocorticoid receptor interacting protein-1, and cAMP response element-binding protein-binding protein, are reported to interact with HNF4α to modulate chromatin [18]. Recruitment of both histone acetyltransferase and deacetylase (HDAC) by HNF4α to the target genes leads to respectively positive and negative regulation of gene expression [19], [20], implicating the dual roles of HNF4α in modulating chromatin for gene expression. In a study integrating protein binding microarrays with chromatin immunoprecipitation coupled with microarrays (ChIP-Chip) and expression profiling, approximately 240 new direct HNF4α target genes were identified [10]. Among these target genes is HDAC6, a class IIb member of HDAC [21]. These previous findings suggest that HNF4α might play a role in the establishment of epigenetic modifications.

At present, many platforms are available for investigation of DNA and histone modifications, including ChIP, N-ChIP, biotin-tag affinity purification and DNA adenine methyltransferase identification for histone modifications [15], as well as bisulfite pyrosequencing, BeadChIP analysis, methylated DNA immunoprecipitation (MeDIP), hydroxymethylated DNA immunoprecipitation (hMeDIP), methyl binding domain, comprehensive high-throughput arrays for relative methylation, luminometric methylation assay, and HpaII tiny fragment enrichment by ligation-mediated PCR for DNA methylation [16]. In ChIP and (h)MeDIP coupled with quantitative PCR, it is usual to calculate a relative enrichment of locus of interest and normalize the estimation with genomic regions which are expectedly unbound to target protein, unmethylated, or unhydroxymethylated. A previous study suggests that the normalization of relative enrichment by the external control in PCR reaction coupled with MeDIP has the advantages in ensuring experimental reproducibility and robustness over the use of internal genomic regions, particularly under the conditions of the specific study [22].

To our best knowledge, no previous studies have been reported to spike external positive and negative controls in ChIP and hMeDIP coupled with qPCR, which are critical to ensure the reproducibility and robustness of the assays by normalizing variations in the processes of immunoprecipitation (IP), preparation of IPed DNA, and/or qPCR quantification. Thus, the aim of the present study was to develop improved ChIP and (h)MeDIP assays by incorporation of spiked DNA fragments as positive and negative controls, and to use the assays to elucidate alterations of hepatic DNA methylation (5 mC and 5 hmC) and representative histone modifications (H3K4me2, H3K4me3, H3K9me2, H3K9me3, H3K27me3 and H3K4ac) in *Hnf4a*-deficient female mice. Our results demonstrate that HNF4**α** is essential in establishment and/or maintenance of epigenetic modifications in liver.

## Materials and Methods

### Preparation of liver samples

The livers of female young-adult mice with liver-specific knockout of *Hnf4a* (*Hnf4a*-LivKO) (*Hnf4a* flox/flox, Alb-cre/+) and age-matched wild-type (*Hnf4a*flox/flox, Alb-cre/-) littermates at the age of 45 days were collected in the previous studies [4] and stored at −80°C until use. All animal procedures in the study were approved by the Institutional Animal Care and Use Committee of the University of Kansas Medical Center [4].

### Positive and negative controls in ChIP, MeDIP, and hMeDIP assays

**Figure 1:**
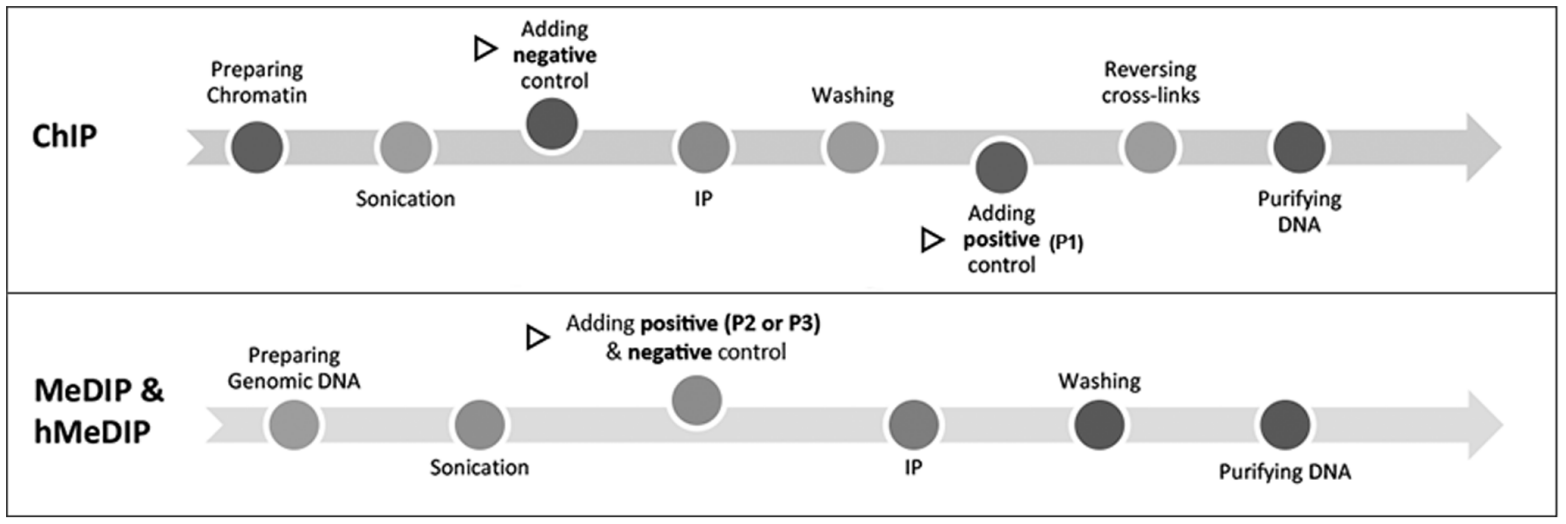
Schematic diagram of the strategy for incorporation of controls in the improved MeDIP, hMeDIP and ChIP. Triangle symbol indicates the step at which the control was introduced.

The external positive (P1, 307 bp) and negative (303 bp) control DNA fragments for the ChIP assay were generated by PCR reactions from pRL-CMV vector (Promega, Madison, WI, USA) (**Fig. 1**, top). The reaction mixture contained 50 ng pRL-CMV vector, 1 µM primers (positive control primers 1, forward: TATGATCCAGAACAAAGGAAACG and reverse: GAAGTTCAAACCATGCAGTAAGA; negative control primers, forward: GGAGAATAACTTCTTCGTGGAAA and reverse: TCAGTATTAGGAAACTTCTTGGC), and JumpStart® PCR master mix (Sigma-Aldrich, St. Louis, MO, USA). The PCR conditions were as follow: 95°C for 10 min; 95°C for 30 s, 60°C for 30 s and 72°C for 30 s for 40 cycles; 72°C for 4 min, in a total volume of 20 µl. Combined PCR products from several tubes were purified with Gel Extraction Kit according to the manufacturer's instructions (OMEGA Bio-Tek, Norcross, GA, USA). The positive control (P2) for MeDIP was derived from P1 methylated by CpG methyltransferase (NEB, Ipswich, MA USA) followed by purification (**Fig. 1**, bottom). The positive control (P3) for hMeDIP was prepared by PCR under the same conditions as those of P1 preparation except that dCTP was replaced by 5-hydroxyl dCTP in the reactions before purification. The quantifications of DNA were performed with Qubit® 2.0 fluorometer (Invitrogen, Grand Island, NY, USA). ChIP, MeDIP, and hMeDIP shared the same negative control. 1 ug each of purified positive control (P1, P2 and P3) was added in a reaction of 50 µl supplemented with 2 units of McrBC which cleaves the methylated and hydroxymethylated DNA, 1× NEB buffer 2, 200 µg/ml BSA and 1 mM GTP, as per manufacturer's instructions. After incubation at 37°C for 1 h, the remaining non-digested controls were quantified by RT-PCR to validate the methylated and hydroxymethylated controls for MeDIP (P2) and hMeDIP (P3). The specificities of detecting primers for positive controls (Primer pairs 2 forward: TGTGCCACATATTGAGCCAGTA and reverse: GAAGTTCAAACCATGCAGTAAGA; Primer pairs 3, forward: TGTGCCACATATTGAGCCAGTA and reverse: ATTACCAGATTTGCCTGATTTGC) and the negative control (forward: GGAGAATAACTTCTTCGTGGAAA and reverse: GCCATGATAATGTTGGACGAC) were assessed by gel electrophoresis after PCR reactions in the presence of human and mouse genomic DNA and input DNA fragments. The primers for mouse *Ugt2b36* promoter (forward: CATGATTTTCCACCAACACAGTA and reverse: GTAATCCATCTGTCACTGCTTG) and human *MDR1* promoter (forward: TGGAGCCATAGTCATGTACTCA and reverse: ACAAGTGTTTATCCCAGTACCA) were used as controls for mouse and human samples, respectively. The PCR conditions for above primers were as follows: 95°C for 10 min; 95°C for 30 s, 60°C for 30 s and 72°C for 30 s for 35 cycles; 72°C for 4 min, in a total volume of 20 µl. The potential mutual interferences between P1 and Input DNA fragments in RT-PCR reaction were also evaluated by comparing one's Cq values in the absence and presence of the other one at low, medium, and high concentrations or dilutions.

### ChIP assay

The ChIP assay was carried out according to the protocol of EZ-Magna ChIP™ A/G Kit (Millipore, Billerica, MA, USA) with minor modifications (**Fig. 1**, top). Briefly, approximately 200 mg of mouse liver were crushed into powder in liquid nitrogen. Liver powders were transferred into centrifuge tubes and incubated with newly prepared 1% formaldehyde in 10 ml PBS for 10 min at room temperature (RT). Crosslinking was quenched by adding 1× glycine and incubation for 5 min at RT. The crosslinked samples were centrifuged at 800 g for 5 min. The supernatant was discarded, and the pellet was washed two times with PBS followed by resuspension in 2 ml cold cell lysis buffer plus 1× protease inhibitor cocktail II, and homogenized with Dounce homogenizer. The homogenate was then centrifuged at 800 g for 5 min at 4°C to precipitate nuclei pellet which was then resuspended in 1 ml nuclear lysis buffer plus 1× protease inhibitor cocktail II. Sonication of nuclear lysates was performed using Sonic Dismembrator (FB-505, Fisher Scientific, Pittsburgh, PA, USA) coupled with Recirculating Chillers (Fisher Isotemp, Pittsburgh, PA, USA) under the following conditions: power of 60%, sonication time of 10 min with pulse on 40 s and pulse off 20 s, temperature set at 2°C. After centrifugation of sonicated lysates at 12000 g at 4°C for 10 min, the supernatant was transferred into a fresh tube. The concentration of sheared chromatin was determined with Qubit® 2.0 fluorometer. 2 µg of chromatin was added into dilution buffer in a total volume of 500 µl. After removal 1% of chromatin as input sample, the antibody, 1× protease inhibitor cocktail II, magnetic beads, and 2 µl negative control (1 pg/µl) were added in the dilution buffer. The antibodies against respective histone modifications are: rabbit monoclonal antibodies against H3K27me3 (C36B11) and H3K9me2 (D85B4) from Cell Signaling Technologies (Danvers, MA, USA), rabbit polyclonal antibody against H3K4me2 (07-030) and mouse monoclonal antibodies against H3K9me3 (05-1242) and H3K4me3 (17-678) from Millipore, rabbit polyclonal antibody against H3K4ac (39381) from Active Motif (Carlsbad, CA, USA). After immunoprecipitation (IP) overnight at 4°C, the magnetic beads were collected with magnetic separator (Magna GrIP™ Rack, Millipore). The beads were washed sequentially with low and high salt wash buffer, LiCl wash buffer, and TE buffer, followed by incubation with elution buffer containing proteinase K and 2 pg of P1 to elute protein/DNA complexes and reverse crosslinks of protein/DNA complexes to release DNA. The DNA fragments were purified by spin columns and dissolved in the elution buffer C of a total volume of 55 µl. The crosslinks of input sample were also reversed in elution buffer containing proteinase K before purification with spin columns. After purification, 2 pg of P1 and 2 pg of negative control were added into input sample to a final volume of 55 µl. DNA concentration was determined by Qubit® 2.0 fluorometer.

### MeDIP and hMeDIP assays

MeDIP and hMeDIP assays were conducted following the protocol described previously [23] with minor modifications (**Fig. 1**, bottom). Briefly, genomic DNA was prepared by phenol, chloroform and isoamylalcohol extraction and then treated with RNase. DNA fragments of 200–600 bp were generated by sonication under the conditions as follows: power of 40%, sonication time of 10 min with pulse on 40 s and pulse off 20 s, temperature set at 2°C. 2 µg of DNA fragments for MeDIP or 10 µg for hMeDIP was added into IP buffer. 1% of fragments in buffer were removed as input sample into a fresh tube. 2 pg of P2 or P3 and 2 pg of negative control were then added into the IP sample and input sample. Mouse monoclonal antibody against 5 mC (clone 33D3, Diagenode, Philadelphia, PA, USA) and rat monoclonal antibody against 5 hmC (MABE176, Millipore) were used to immunoprecipitate methylated and hydroxymethylated DNA fragments, respectively, in IP buffer with Dynabeads® M-280 sheep anti-mouse IgG beads or anti-rat IgG beads (Invitrogen). After 2 h incubation at 4°C, the beads were collected and resuspended in IP buffer plus proteinase K for incubation at 50°C for 3 h to digest proteins. The recollected beads were discarded and the supernatant was transferred to a fresh tube for purification with Gel Extraction Kit (Omega Bio-Tek). The concentration of resulting DNA fragments was determined by Qubit® 2.0 fluorometer.

### RNA isolation and cDNA preparation

Total RNA was extracted by using RNA STAT-60 (Tel-Test, Friendswood, TX, USA). Briefly, frozen liver tissues (n = 4) were homogenized in RNA STAT-60, and RNA was precipitated by the addition of isopropanol. After washing with 75% ethanol, the RNA pellet was air-dried and dissolved in RNase-free water. cDNA was produced by the use of High Capacity cDNA Reverse Transcription Kit (Applied Biosystems, Foster City, CA, USA) according to the manufacturer's instructions. The resultant cDNA was used as the templates for RT-PCR reactions.

### RT-PCR quantification of enriched DNA fragments and transcripts

The enriched DNA fragments in ChIP, MeDIP, and hMeDIP were quantified with RT-PCR reaction containing DNA fragments, 500 nM primers (sequences in **Table S3 in File S1**) and iQ™ SYBR® Green Supermix (Bio-Rad, Hercules, CA, USA) by MyiQ2™ Two-Color Real-Time PCR Detection System (Bio-Rad). The PCR conditions, unless otherwise specified in **Table S3 in File S1**, were as follows: 95°C for 3 min; 95°C for 15 s, 60°C for 30 s and 72°C for 30 s for 40 cycles in a total volume of 10 µl. Percentage of enrichment of epigenetic signatures at loci of interest normalized by positive control was calculated based on the following formula: 

Where “E” is the efficiency of amplification in RT-PCR, “target” is the locus of interest and “positive” is each positive control (normalizer) for ChIP, MeDIP, and hMeDIP.

The prepared cDNA from mouse livers was quantified by RT-PCR under the same conditions as IPed samples. Amounts of mRNAs were calculated using the comparative CT method, which determines the amount of target normalized to β-actin, with the wild-type control value set at 1.0. Each Cq value used for the calculation was the mean of triplicates.

### Preparation of histones assembled to chromatin and Western Blot quantification of histones

Nuclear fractions of histones were prepared as reported previously [24], [25] with minor modification. Briefly, the mouse livers were homogenized in hypotonic buffer (20 mM HEPES.KOH (pH 7.8), 5 mM potassium acetate, 0.5 mM MgCl_2_ and protease inhibitor). After centrifugation at 1500g for 5min, the supernatant was removed. The pelleted nuclei were incubated at 4°C with gentle mixing for 1h in PBS supplemented with 0.1% Triton X-100. The DNA-bound and –unbound histones were fractionated by centrifuging at 12000g for 10min. The resultant pellets were incubated with RIPA buffer followed by centrifugation to release the histones from chromatin. Histones in the resultant supernatant were resolved in sodium dodecyl sulphate-polyacrylamide gel electrophoresis (SDS-PAGE). Western blot quantification of histones was carried out with the primary antibodies same as those in ChIP assay and the anti-H3 antibody (D1H2) from Cell Signaling Technologies. Primary antibodies were revealed with HRP-conjugated secondary antibodies (anti-rabbit IgG (W4011, Promega) or anti-mouse IgG (A4416, Sigma)) and ECL Western Blotting Substrate (W1015, Promega). ChemiDoc™ XRS+ System (Bio-Rad) and Image Lab 4.0 software (Bio-Rad) were used to capture signals and determine signal intensities.

### Statistical analysis

All values are expressed as mean ± S.E. The student's t-test was used to determine the statistical difference between *Hnf4a*-LivKO and wild-type samples (SigmaPlot 12.0). Statistical significance was set at p<0.05.

## Results

### Preparation and validation of the external controls

**Fig. 1** is the schematic diagram illustrating the strategy for the incorporation of external controls in the present MeDIP, hMeDIP and ChIP assay. Given that in ChIP it is technically unfeasible to add positive and negative controls simultaneously in the beginning of IP, we added the negative and positive controls before and after IP, respectively. The negative control reflects the nonspecific binding of DNA to tube and reaction substances such as magnetic beads and antibody in dilution buffer, and the positive control serves as a normalizer to compensate for the deviations in the ChIP procedures of elution and purification as well as the following RT-PCR reactions.

**Figure 2:**
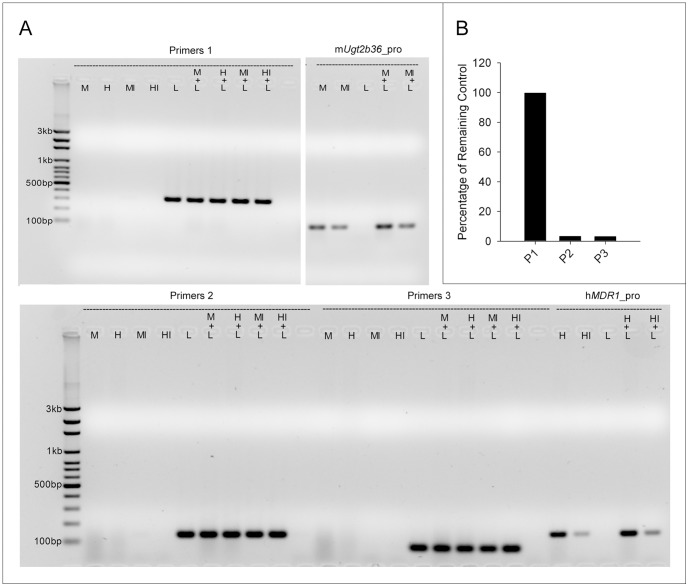
Specificities of positive control primers and validation of positive controls employed in MeDIP and hMeDIP assays. A: Gel electrophoresis of PCR products of primer pairs 1, 2, and 3 for positive control fragments in the presence/absence of mouse genomic DNA (M), human genomic DNA (H), mouse input DNA (MI), human input DNA (HI), and pRL-CMV control template (L). Human genomic and input DNA samples were prepared from HepG2 cells (ATCC). The primers for mouse *Ugt2b36* promoter and human *MDR1* promoter were selected as control primers. B: Validation of methylated (P2) and 5-hydroxymethylated (P3) positive control fragments used in MeDIP and hMeDIP, respectively, by McrBC endonuclease digestion. 1 ug each of purified positive control (P1, P2 and P3) was added in a reaction of 50 µl supplemented with 2 units of McrBC which cleaves the methylated and hydroxymethylated DNA, 1× NEB buffer 2, 200 µg/ml BSA and 1 mM GTP, as per manufacturer's instructions. After incubation at 37°C for 1 h, the remaining non-digested controls were quantified by RT-PCR to validate the methylated and hydroxymethylated controls for MeDIP (P2) and hMeDIP (P3). P1 is the unmethylated control used in the ChIP assay.

The positive and negative controls in ChIP, MeDIP, and hMeDIP assays were generated by PCR amplification of fragments of Renilla luciferase cDNA using the vector pRL-CMV (Promega) as a template to avoid the homologies to human and mouse genomic DNA, the context in which the assays will be carried out. The lengths of the positive controls and negative control are 307 and 303bp, respectively, which are the average sizes of sonicated DNA fragments in three assays. Gel electrophoresis in **Fig. 2A** shows that 3 pairs of primers for the positive control P1 did not generate any nonspecific bands for the human and mouse genomic and fragmented DNA input samples, but specifically primed the PCR products for target templates (positive control). The three pairs of primers for positive controls generated PCR products of 307, 134, and 79bp, respectively, which allows for the selection of one primer pair used in regular PCR followed by gel electrophoresis so that the resulting PCR products of both the positive control and the target gene locus can be visualized and analyzed simultaneously. Primer pair 2 was selected in the present study to quantify positive controls in real-time PCR (RT-PCR) after ChIP, MeDIP, and hMeDIP. The specificity of the negative control was also assessed with electrophoresis demonstrating that the primer pair was specific for the negative control in the context of human and mouse samples (Data not shown).

The positive control P2 and the positive control P3 were generated by the methylation of P1 and PCR reaction for MeDIP and hMeDIP, respectively. To validate P2 and P3, the controls were digested by McrBC endonuclease which can cleave methylated and hydroxymethylated DNA but not unmethylated DNA. In contrast to the unmethylated P1 (positive control for ChIP) that had no degradation after digestion, more than 97% of P2 and P3 were destroyed as shown in **Fig. 2B**, indicating that the positive controls for MeDIP and hMeDIP were successfully prepared.

The amplification efficiencies in the RT-PCR reactions for all positive and negative controls were derived from the standard plots each generated with 5 orders of magnitude ranging from 0.01fg to 100 fg. The values of PCR efficiency were 1.93, 1.83, 2.01, and 1.93 for P1, P2, P3, and negative controls, respectively, demonstrating that it is suitable to quantify P1, P2, and P3 by using the same pair of primers (primers 2). The final amounts of 3 positive and 1 negative controls used in RT-PCR were approximately 40 fg, which resulted in Cq values around 20. The ratio of added controls relative to the total DNA fragments in the IP step was less than 1∶10^6^, which is beneficial for downstream analysis such as microarray and deep sequencing. The potential interference of the positive control with the determination of target locus exemplified by UDP glucuronosyltransferase 2 family, polypeptide b36 (*Ugt2b36*) promoter in the context of mouse liver DNA fragments was evaluated by comparing the Cq values of *Ugt2b36* promoter in the presence/absence of the positive control. As illustrated in **Table S1 in File S1**, the positive control did not have a significant effect on the RT-PCR reaction of *Ugt2b36* promoter. The efficiencies were consistent across four groups with values of 1.91–1.96. Likewise, the existence of mouse genomic DNA fragments at all dilution factors did not affect the PCR efficiencies of the positive control, with efficiency values of 1.90–1.94. In addition, the coefficient of variation (CV) values of Cq for all groups were less than 2%, indicating that the RT-PCR system worked well and could be utilized for subsequent quantitative experiments.

The enrichment ratio of positive to negative control was about 600∶1 and 1800∶1 in MeDIP and hMeDIP assay, respectively, further confirming the validity of our methylated (P2) and hydroxymethylated (P3) controls. The CV values of P2 and P3 enrichment in six technical replicates were more than 20%, underscoring the technical deviations from the procedures and the importance of incorporation of an external control to normalize the MeDIP and hMeDIP assays. The average CV values for loci of interest in 3 technical replicates were decreased from 21% and 41% to 13% and 17% for MedIP and hMeDIP, respectively, after the normalization by the corresponding positive control.

In ChIP assay, the enrichment for negative control was less than 0.02% and the positive control was recovered more than 43%. P1 in ChIP had a relatively lower CV of recovery equal to 17.5%, compared to P2 and P3 in MeDIP and hMeDIP, accounting for the technical deviations from the procedures after the washing step in ChIP. The average CV value for loci of interest in 3 technical replicates was slightly decreased, from 25% to 21%, after the normalization by the positive control.

### Effect of *Hnf4a* deficiency on DNA methylation and 5-hydroxymethylation in female mouse livers

**Figure 3:**
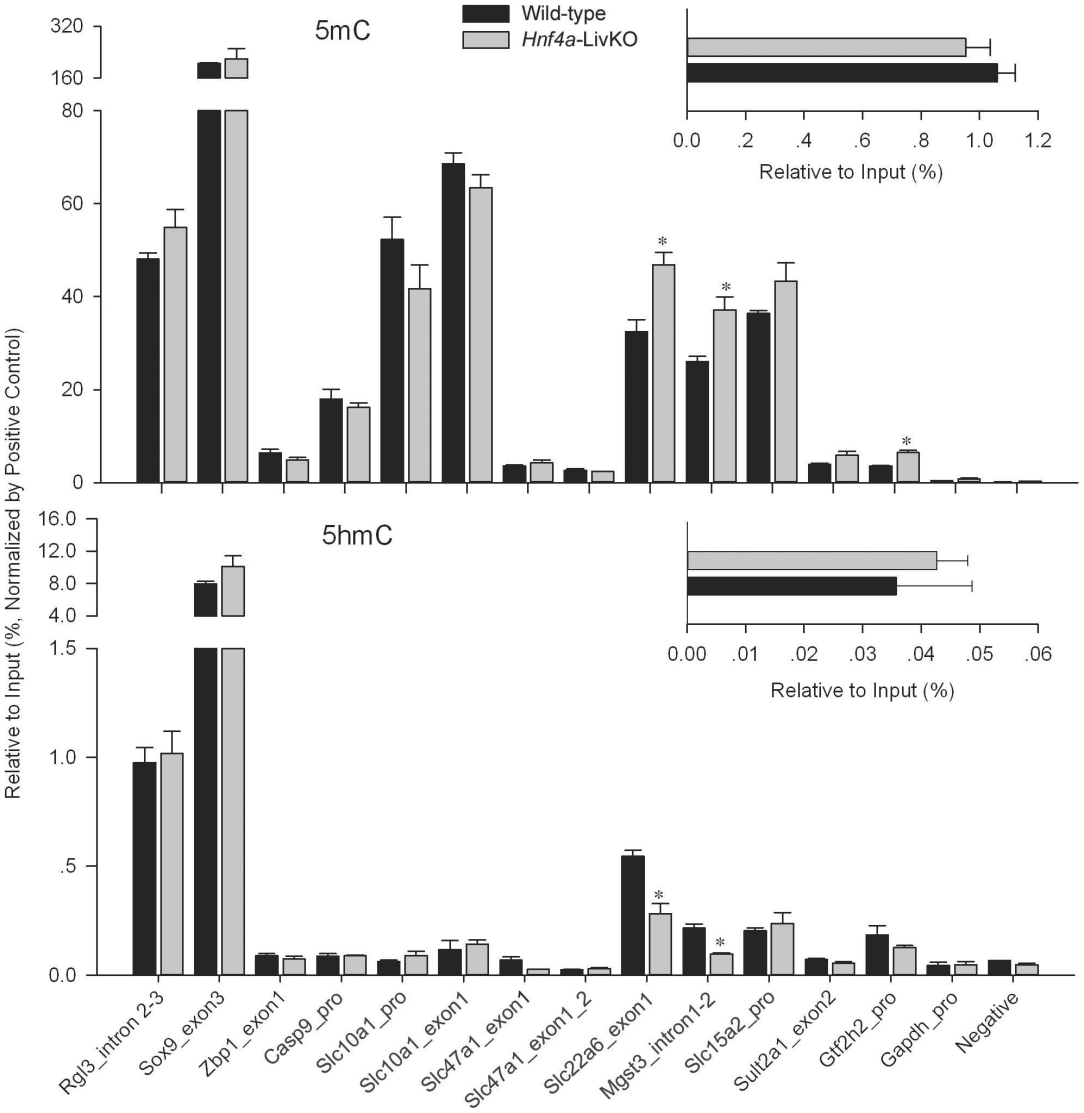
Effects of *Hnf4a* deficiency on DNA methylation (5 mC) and 5-hydroxymethylation (5 hmC) in female mouse livers. Enrichment of 5*Hnf4a*-LivKO and wild-type mice. Insets, the percentage of total (h)MeDIPed DNA fragments relative to input for 5 mC and 5 hmC from *Hnf4a*-LivKO and wild-type mouse livers. Negative = negative control. Mean ± S.E., N = 3 biological replicates. *, p<0.05 versus wild-type.

Having prepared and validated the positive and negative controls, we applied these controls in MeDIP and hMeDIP to quantify the enrichment of loci of interest in liver samples from female young-adult *Hnf4a*-LivKO mice and wild-type littermates. We interrogated the particular loci of enrichment normalized by the positive control in MeDIPed and hMeDIPed samples. The loci chosen for determination were based on their potentially alterable 5 mC and 5 hmC status during development that was proven or speculated by previous studies [26], [27]. For example, DNA methylation in the promoter of Na+-taurocholate cotransporting polypeptide (*Slc10a1*/*Ntcp*) and H+/peptide transporter 2 (*Slc15a2*/*Pept2*) is strongly negatively associated with their tissue-specific expression in liver and kidney [26]. Results from MeDIP-Chip demonstrate that the loci in intron2–3 of ral guanine nucleotide dissociation stimulator-like 3 (*Rgl3*) and exon3 of SRY-box containing gene 9 (*Sox9*) have much higher 5 mC in adult mouse livers than in embryonic mouse livers. Conversely, the loci in promoter of *Caspase 9* (*Casp9*) and exon1 of Z-DNA binding protein 1 (*Zbp1*) have much higher 5 mC in embryonic mouse livers than adult mouse livers [27]. The insets of **Fig. 3** show that the percentage of the total IPed DNA fragments relative to input in wild-type mouse livers was 1.06% for MeDIP and 0.036% for hMeDIP. There were no significant differences in the total IPed fragment amounts between *Hnf4a*-LivKO and wild-type livers, indicating that *Hnf4a* deficiency in young-adult mice does not extensively affect DNA methylation and hydroxymethylation. For specific loci, *Rgl3* intron2-3 and *Sox9* exon3 had higher 5 mC than *Casp 9* promoter and *Zbp1* exon1 in wild-type mouse livers. Nevertheless, *Hnf4a* deficiency had no significant effects on 5 mC and 5 hmC of these four loci (**Fig. 3**). The genes including *Slc10a1*, multidrug and toxin extrusion protein 1 (*Slc47a1*/*Mate1*), organic anion transporter 1 (*Slc22a6*/*Oat1*), microsomal glutathione *S*-transferase 3 (*Mgst3*), *Slc15a2*, and sulfotransferase 2a1 (*Sult2a1*) encode enzymes and transporters responsible for drug metabolism and disposition in the liver and kidney [4], [28]. General transcription factor II H, polypeptide 2 (*Gtf2h2*) encodes TFIIH which has a central role in the transcription of RNA polymerases [29]. *Hnf4a* deficiency did not affect the 5 mC and 5 hmC of loci in *Slc10a1* promoter and exon1, *Slc47a1* exon1, *Slc15a2* promoter, and *Sult2a1* exon2, as well as the promoter of the housekeeping gene glyceraldehyde-3-phosphate dehydrogenase (*Gapdh*). In contrast to the above gene sites, the loci in *Slc22a6* exon1, *Gtf2h2* promoter, and *Mgst3* intron1-2 were methylated at higher levels in *Hnf4a*-LivKO livers than those in wild-type livers. Intriguingly, the 5 hmC of *Slc22a6* exon1 and *Mgst3* intron1–2 were decreased accordingly due to *Hnf4a* deficiency. *Gtf2h2* promoter displayed slight but insignificant decrease of 5 hmC in *Hnf4a*-LivKO Liver (**Fig. 3**).

### Effect of *Hnf4a* deficiency on histone modifications in female mouse livers

**Figure 4:**
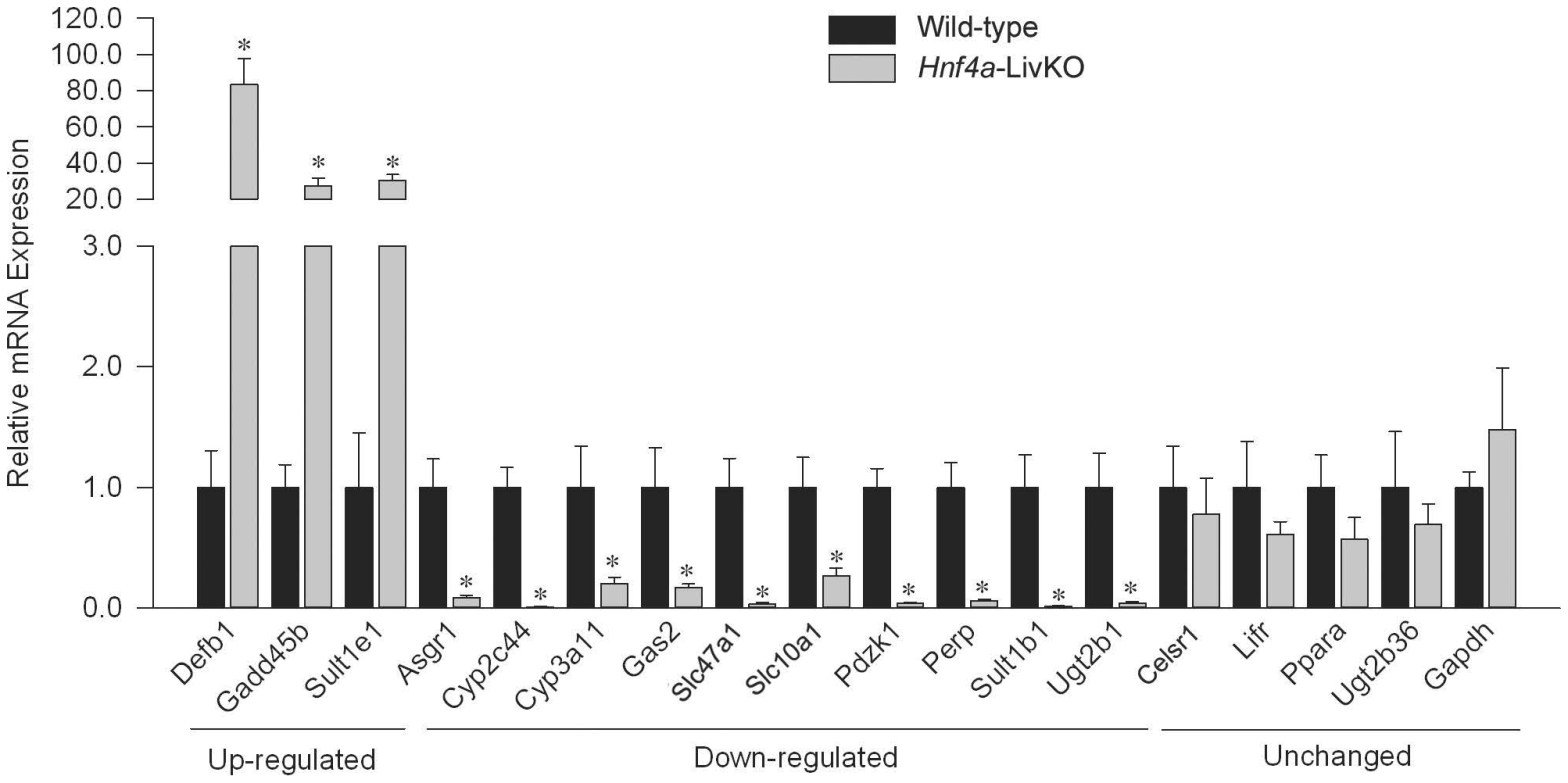
Real-time PCR quantification of mRNA expression in livers from female *Hnf4a*-LivKO and wild-type mice. The y-axis represents relative mRNA expression with values of wild-type set at 1.0. The genes investigated are categorized into three groups, namely up-regulated, down-regulated, and unchanged. Mean ± S.E., N = 4 biological replicates. *, p<0.05 versus wild-type.

To elucidate the effect of *Hnf4a* deficiency on histone modifications, we determined the enrichment of H3K4me2, H3K4me3, H3K9me2, H3K9me3, H3K27me3, and H3K4ac in a set of gene loci. The list of tested genes and their main functions are illustrated in **Table S2 in File S1**. All these genes, except *Gapdh*, are HNF4á-dependent genes (either up-regulated or down-regulated) determined in previous studies by Quantigene Plex assay or microarray [4], [18] that employed the same *Hnf4a*-LivKO model as ours in the present study. The proteins encoded by the selected genes are important in HNF4α-regulated processes in liver, such as xenobiotic metabolism, cellular defense, or apoptosis. Among these genes, *Perp* has been reported as a direct HNF4α-target gene in mouse liver [5], and *PDZK1* is also a direct HNF4α-target gene in human cell line [10]. We first determined hepatic changes in mRNA expression of these genes in *Hnf4a*-deficient female mouse livers (**Fig. 4**). *Hnf4a* deficiency markedly induced mRNA expression of *Defb1*, *Sult1e1*, and *Gadd45β* (up-regulated group), but down-regulated *Asgr1*, *Cyp2c44*, *Cyp3a11*, *Gas2*, *Slc47a1*, *Slc10a1*, *Pdzk1*, *Perp*, *Sult1b1*, and *Ugt2b1* (down-regulated group). For other genes *Celsr1*, *Lifr*, *Ppara*, and *Ugt2b36* (unchanged group), *Hnf4a* deficiency slightly but did not significantly decrease their mRNA expression. The trends of alterations of gene expression due to *Hnf4a* deficiency are generally in agreement with the previous data [4], [8], [18].

**Figure 5:**
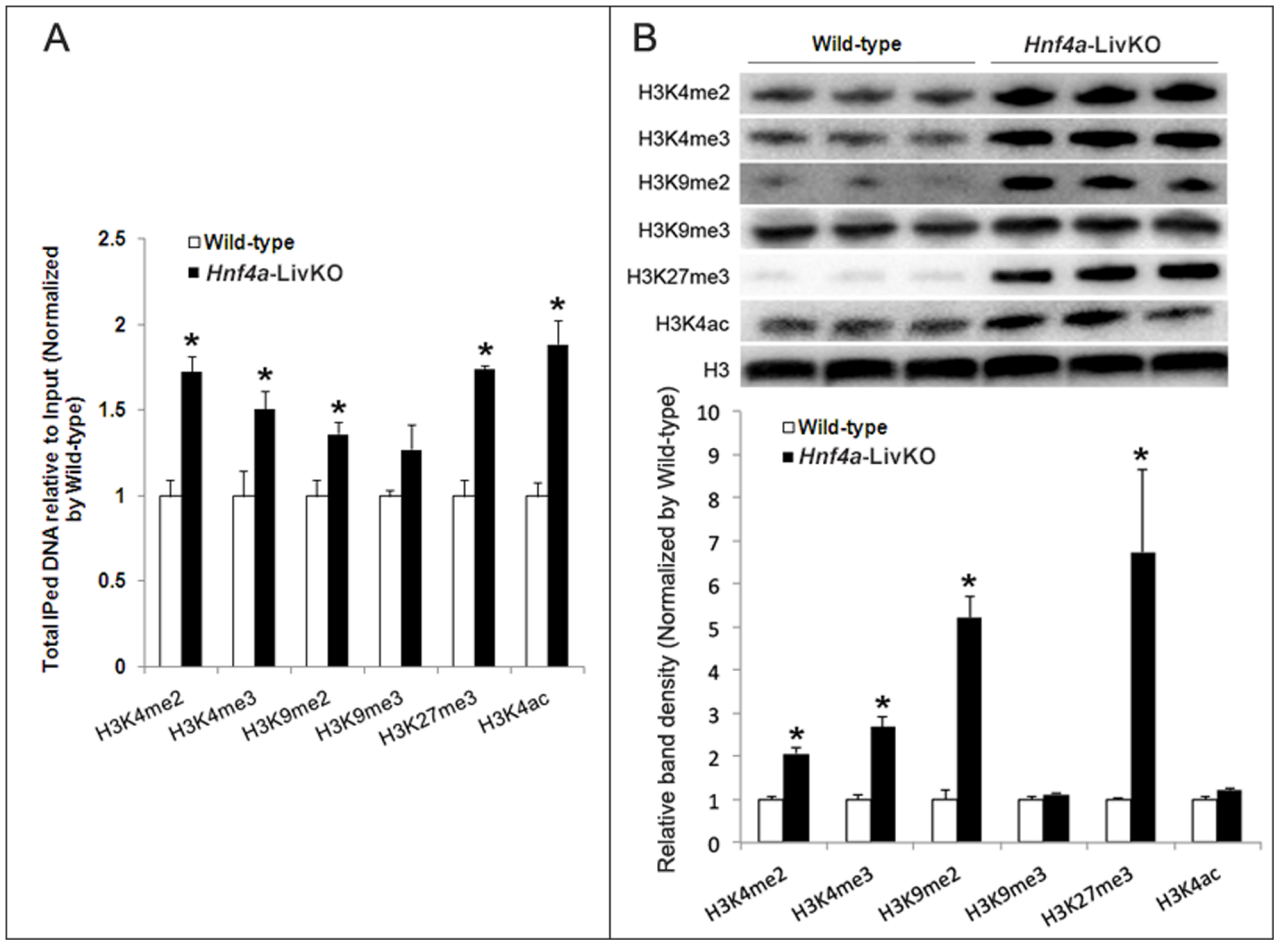
Global alterations of histone H3 lysine-4 dimethylation (H3K4me2), H3K4me3, H3K9me2, H3K9me3, H3K27me3 and H3K4ac in female wild-type and *Hnf4a*-LivKO mouse livers. A: The total ChIPed DNA fragments relative to input for 6 histone modifications in livers from female *Hnf4a*-LivKO and wild-type mice. B: Representative Western blot and band density analyses of H3K4me2, H3K4me3, H3K9me2, H3K9me3, H3K27me3 and H3K4ac in histones assembled to chromatin from livers of female *Hnf4a*-LivKO and wild-type mice. Mean ± S.E., N = 3 biological replicates. *, p<0.05 versus wild-type.

We next determined global and specific alterations of the representative histone modifications in *Hnf4a*-LivKO and wild-type mouse livers. As shown in **Fig. 5A**, *Hnf4a* deficiency significantly increased the total ChIPed DNA fragments by 73%, 51%, 36%, 74%, and 89% for H3K4me2, H3K4me3, H3K9me2, H3K27me3 and H3K4ac, respectively, compared with wild-type. In contrast, the total enrichment for H3K9me3 was not significantly changed. To validate the findings in ChIP, we further used Western blot to quantify these histone marks in DNA-bound histones prepared from wild-type and *Hnf4a*-LivKO mouse livers. We used 0.1% Triton to remove the soluble histones to prepare insoluble histones (assembled to chromatin) which are the targets of antibodies in ChIP assay. In general, the alterations of the histone modifications in *Hnf4a*-LivKO from Western blot are consistent with those of total ChIPed fragments in ChIP. The total DNA-bound H3K4me2, H3K4me3, H3K9me2, and H3K27me3 in *Hnf4a*-LivKO mice were 2.1, 2.7, 5.2, and 6.7 fold higher, respectively, than those in wild-type mice (**Fig. 5B**). Similar to ChIP, Western blot data showed that the total DNA-bound H3K9me3 was not significantly altered by *Hnf4a* deficiency. For H3K4ac, *Hnf4a*-LivKO mice had significantly higher total ChIPed DNA fragments, but only slightly higher (20%, non-significant) total DNA-bound H3K4ac than wild-type mice (**Fig. 5B**). The above results demonstrate that *Hnf4a* deficiency markedly affects most histone modifications investigated herein.

Next we interrogated the enrichment of each histone modifications tested at specific loci. The results are summarized in **Table 1**. Most loci (just one exception, *Gas2* promoter) harbored in the down-regulated genes had more enrichment of H3K4me2 in *Hnf4a*-LivKO mouse livers than in wild-type livers. However, all 3 up-regulated genes (*Defb1*, *Sult1e1*, and *Gadd45*β) had no changes in H3K4me2 at the promoter. Unchanged genes including *Celsr1*, *Ppara*, and *Ugt2b36* also had more H3K4me2 in their promoters in *Hnf4a*-LivKO livers.

**Table 1:** Effects of *Hnf4a* deficiency on H3K4me2, H3K4me3, H3K9me2, H3K9me3, H3K27me3 and H3K4ac at the specific loci in female mouse livers determined by the improved ChIP-qPCR.

		Histone modifications^a^					
Locus	Group^b^	H3K4me2	H3K4me3^c^	H3K9me2	H3K9me3	H3K27me3	H3K4ac
*Defb1*_pro	↑	–	1.5	–	–	–	–
*Gadd45b*_pro	↑	–	1.8	–	–	–	–
*Sult1e1*_pro	↑	–	–	–	–	–	1.6
*Asgr1*_pro	↓	2.0	–	1.7	–	2.5	1.7
*Cyp2C44*_pro	↓	1.5	–	1.5	1.7	2.0	1.7
*Cyp3a11*_pro	↓	2.0	–	–	–	1.7	1.6
*Gas2*_pro	↓	–	–	–	–	2.0	1.7
*Slc47a1*-exon1	↓	1.8	–	3.3	–	2.8	–
*Slc10a1*_pro	↓	1.8	0.6	2.6	–	1.6	1.4
*Pdzk1*_pro	↓	1.5	0.6	1.8	–	1.8	1.7
*Perp*_pro	↓	1.7	0.6	1.8	–	2.6	1.7
*Sult1b1*_pro	↓	1.8	–	1.4	–	2.0	2.1
*Ugt2b1*_pro	↓	1.8	–	1.9	–	–	1.7
*Celsr1*_pro	–	2.5	–	–	–	1.6	1.4
*Lifr*_pro	–	–	–	2.5	–	1.9	1.4
*Ppara*_pro	–	1.7	–	1.6	–	3.0	1.9
*Ugt2b36*_pro	–	1.6	–	1.9	2.1	–	1.6
*Gapdh*_pro	–	–	–	–	–	–	–

^a^ The enrichment of histone modification at the specific locus (relative to input) in livers of *Hnf4a*-LivKO is divided by that in wild-type to calculate the fold of alteration. The number signifies the fold of alteration that is significant in *Hnf4a*-LivKO mice (p <0.05 vs. wild-type mice). “–” signifies insignificant alteration in *Hnf4a*-LivKO mice. **Fig. S1**, **Fig. S2** and **Fig. S3 in** **File S1** show the same data in the column plots.

^b^ The genes harboring loci are categorized into same groups as those in **Fig. 4**: Up-regulated (↑), down-regulated (↓) and unchanged (–), which are separated by dotted lines.

^c^ Additional loci for H3K4me3 were tested, including *Cyp2c44* exon1, *Cyp2c44* exon2, *Sult1b1* exon1, *Sult1b1* exon2, *Sult1e1* exon2, *Ugt2b1* exon1 and *Ugt2b1* exon2. Among these loci, the alterations at *Cyp2c44* exon1, *Ugt2b1* exon1 and *Ugt2b1* exon2 are significant, with the fold of 0.2, 0.5 and 0.3, respectively.

The enrichment of H3K4me3 in the gene loci investigated was affected to a lesser extent by *Hnf4a* deficiency than that of H3K4me2. At the same loci, only those in the promoters of *Defb1*, *Gadd45β*, *Slc10a1*, *Pdzk1*, and *Perp* had changes in enrichment of H3K4me3 in *Hnf4a*-LivKO livers. Intriguingly, the loci harbored in up-regulated genes *Defb1* and *Gadd45β* had increased, whereas the loci in down-regulated genes such as *Slc10a1* and *Pdzk1* had decreased enrichment of H3K4me3 due to *Hnf4a* deficiency. In addition, we also determined the enrichment of H3K4me3 in additional loci located in the gene body, including *Cyp2c44*, *Sult1b1*, *Sult1e1*, and *Ugt2b1* exons. *Cyp2c44* exon1 but not exon2 as well as *Ugt2b1* exon1 and exon2 had significantly lower H3K4me3 enrichment in *Hnf4a*-LivKO than in wild-type livers, although the promoters of these genes had no changes in H3K4me3. In contrast, *Hnf4a*-LivKO mouse livers had no changes in H3K4me3 in exon1 and exon2 of *Sult1b1* or exon 2 of *Sult1e1*, similar to those in the promoters of *Sult1b1* and *Sult1e1*.

The enrichment of H3K9me2 in most down-regulated and unchanged loci was increased by *Hnf4a* deficiency, whereas the up-regulated loci were not affected. In contrast, H3K9me3 was not changed in the majority of loci investigated, with the exception of increases in the *Cyp2c44* and *Ugt2b36* promoters in *Hnf4a*-LivKO mice.

Most of the down-regulated and unchanged genes, except *Ugt2b1* and *Ugt2b36*, were enriched more for H3K27me3 in *Hnf4a*-LivKO than in wild-type livers, whereas the up-regulated gene loci were not affected. In regard to H3K4ac, *Hnf4a* deficiency increased its enrichment at the majority of tested loci regardless of up-, down-regulated, or unchanged genes, with the exception of loci in the *Defb1* promoter, the *Gadd45β* promoter, and *Slc47a1* exon1. The promoter of housekeeping gene *Gapdh* was used as a negative reference, which expectedly had no changes in the six histone modifications in *Hnf4a*-LivKO mice. Collectively, *Hnf4a* deficiency affects H3K4me2, H3K4me3, H3K9me2, H3K9me3, H3K27me3 and H3K4 acetylation, to different degree, at the loci of these tested genes.

### Effect of *Hnf4a* deficiency on hepatic mRNA expression of epigenetic modifiers

**Figure 6:**
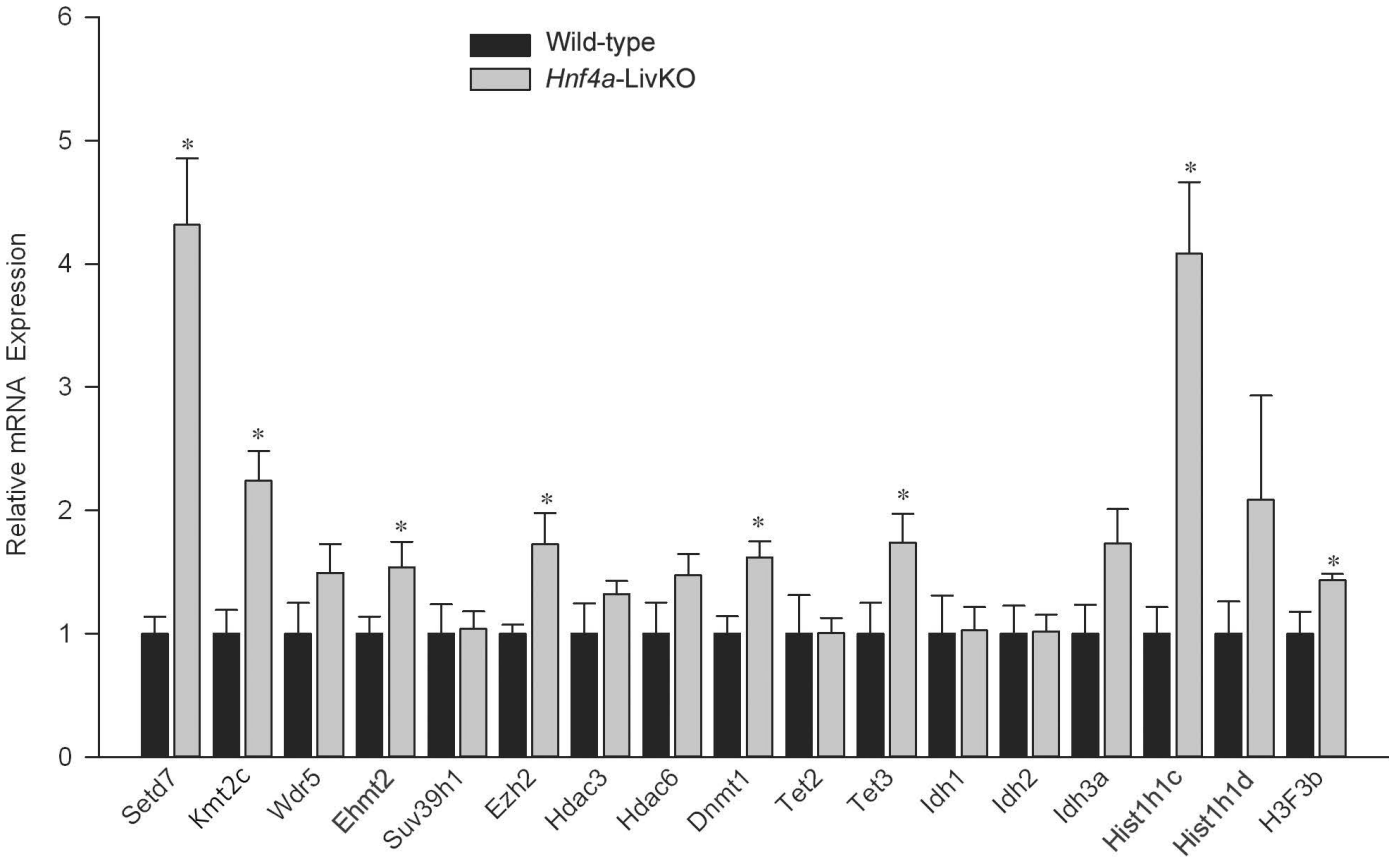
Real-time PCR quantification of mRNA expression of genes encoding epigenetic modifiers and histones in livers from female *Hnf4a*-LivKO and wild-type mice. The Y-axis represents relative mRNA expression with values of wild-type set at 1.0. Mean ± S.E., N = 4 biological replicates. *, p<0.05 versus wild-type.

To elucidate the mechanism of the multiple changes in hepatic epigenetic signatures due to *Hnf4a* deficiency, we determined the alteration of mRNA expression of genes encoding a set of epigenetic modifiers in the *Hnf4a*-LivKO mouse liver (**Fig.6**), including SET domain containing 7 (*Setd7*), mixed-lineage leukemia 3 (*Kmt2c*/*Mll3*), WD repeat domain 5 (*Wdr5*), euchromatic histone lysine N-methyltransferase 2 (*Ehmt2*/*G9a*), suppressor of variegation 3-9 homolog 1 (*Suv39h1*), enhancer of zeste homolog 2 (*Ezh2*), histone deacetylase 3 (*Hdac3*), Hdac6, DNA methyltransferase (cytosine-5) 1 (*Dnmt1*), tet methylcytosine dioxygenase 2 (*Tet2*), *Tet3*, isocitrate dehydrogenase 1 (*Idh1*), *Idh2*, and *Idh3a* which are important for dynamically laying down and/or removing modifications to DNA and histones [13], [30]–[34]. *Hnf4a* deficiency significantly induced mRNA expression of *Setd7*, *Kmt2c*, *Ehmt2*, *Ezh2*, *Dnmt1*, and *Tet3*, but not *Wdr5*, *Suv39h1*, *Hdac3*, *Hdac6*, *Tet2*, *Idh1*, *Idh2*, and *Idh3a*. In addition, the expression of *Hist1h1c* encoding H1.2 [35] and *H3f3b* encoding H3.3 [36] histone was induced, whereas the expression of *Hist1h1d* encoding H1.3 [35] was not altered in *Hnf4a*-LivKO livers (**Fig. 6**), suggesting that *Hnf4a* possibly plays a role in the regulation of the histone H1 isoform and H3.3 variant.

## Discussion

In the present study, we successfully developed the improved MeDIP-, hMeDIP-, and ChIP-qPCR assays to elucidate the impact of *Hnf4a* deficiency on the histone modifications as well as DNA methylation and 5-hydroxymethylation in the female mouse livers. *Hnf4a* deficiency markedly alters histone modifications including H3K4me2, H3K4me3, H3K9me2, H3K27me3 and H3K4ac, whereas its impacts on H3K9me3 and DNA methylation are not as extensive as the preceding modifications. Western blot analyses of the histone modifications further confirm the findings in the ChIP assay. Concomitant to the increase in DNA methylation at certain loci, 5-hydroxymethylation of the corresponding loci decreases due to *Hnf4a* deficiency. The marked changes in hepatic epigenetic signatures in *Hnf4a*-LivKO mice are associated with changes in hepatic mRNA expression of epigenetic modifiers.

To elucidate the epigenetic mechanism of regulation of hepatic gene expression by HNF4α, we first established validated external controls for MeDIP-, hMeDIP-, and ChIP-qPCR assays to normalize the variations introduced during the assays. A previous study suggests that conventional housekeeping genes may not be an optimal normalizer for enrichment calculation in MeDIP assay because the methylation status of these genes may be altered under certain conditions [22]. Currently, several commercial MeDIP kits also contain control fragments to normalize the enrichment values and validate the assay procedures [16]. Although bisulfite sequencing was initially considered the most precise method for methylome study [15], subsequent reports indicate that bisulfite method is incapable of distinguishing 5 mC from 5 hmC [37]. As such, MeDIP specific for 5 mC and hMeDIP specific for 5 hmC using specific antibodies against 5 mC or 5 hmC are relatively optimal methods to interrogate the (hydroxy) methylation status at a specific gene locus or genome-wide scale. The negative and positive controls developed in this study provide precise and unbiased normalization of enrichment in MeDIP, hMeDIP, and ChIP, and the convenience in carrying out assays with the reagents prepared in the laboratory. Although spin columns are used in the present study to purify the IPed DNA fragments, it is rationally speculated that our controls can be readily used in procedures with other purification methods such as the collection with magnetic beads. The results of the relatively large deviations of positive controls in technical replicates in MeDIP, hMeDIP, and ChIP indicate that it is important to incorporate external control fragments in these assays to normalize the quantification of relative enrichment of target loci in RT-PCR, which may be followed by microarray and genome-wide sequencing analysis.

The present study demonstrates that *Hnf4a* deficiency in young-adult mouse liver does not cause global changes in DNA methylation and 5-hydroxymethylation, but produces specific changes in certain gene loci. Even if *Hnf4a* deficiency does not change the amount of total methylated DNA fragments, the methylation of *Slc22a6* exon1 and *Mgst3* intron 1–2 increase in *Hnf4a*-LivKO livers, which is associated with the increased mRNA expression of *Dnmt1*. *Dnmt1* is a maintenance DNA methyltransferase that predominantly methylates hemimethylated CpG dinucleotides. Most interestingly, the 5-hydroxymethylation at these two loci decreases correspondingly in *Hnf4a*-LivKO livers, suggesting the conversion of 5 hmC to 5 mC due to *Hnf4a* deficiency. Most recently, Lian and colleagues reported that loss of 5 hmC is an epigenetic hallmark of melanoma, and IDH2 and TET family are directly linked to this process [30]. Our findings that mRNA expression of *Tet2* and *Idh 1*, *2* and *3a* is not changed, whereas *Tet3* expression is increased in *Hnf4a*-LivKO livers does not support the roles of *Tet* and *Idh* in the decreases of 5 hmC at the gene loci in *Hnf4a*-LivKO livers.

*Hnf4a* deficiency in young-adult mouse liver causes a global increase in H3K4me2. In general, histone acetylation and H3K4 methylation are associated with transcribed chromatin, and methylation of H3K9 and H3K27 correlates with gene repression [13], [38], [39]. However, previous studies also suggest that H3K4me2 is implicated in transcriptional repression [40], and H3K9me3 is within a number of actively transcribed regions [15]. The present data support the repressive role of H3K4me2 in gene regulation in that increases of H3K4me2 correspond to the down-regulated and unchanged, but not up-regulated genes, which is similar to the repressive marks of H3K9me2 and H3K27me3.

*Hnf4a* deficiency in young-adult mouse liver does not cause global change in H3K9me3. H3K9me3 is associated with heterochromatin which is a tightly packed form of DNA [41]. The alterations of H3K9me3 due to *Hnf4a* deficiency are not as extensive as the other five tested histone modifications based on the data of total ChIPed DNA fragments, the Western blot data, and the enrichments at specific gene loci, suggesting that selective condensing of a certain gene locus might be a mechanism underlying highly silenced genes such as Cyp2c44.

*Hnf4a* deficiency in young-adult mouse liver causes a global increase in H3K9me2. Methyltransferase *Ehmt2* is capable of mono, di-, and trimethylation of H3K9, whereas Suv39h1 and Suv39h2 are largely responsible for H3K9 trimethylation [42]. *Ehmt2* is induced, whereas Suv39h1 is not affected by *Hnf4a* deficiency, which is consistent with the increased total H3K9me2 but unchanged total H3K9me3 in *Hnf4a*-LivKO mouse livers. These results suggest that the regulation of H3K9me2 by HNF4α in the down-regulated and unchanged gene loci may be mediated, at least partly, by *Ehmt2*.

*Hnf4a* deficiency in young-adult mouse liver causes a global increase in H3K27me3. Ezh2 is the methyltransferase responsible for trimethylation of H3K27 [32], which is also associated with heterochromatin [41] as is H3K9me3. Induction of Ezh2 in *Hnf4a*-LivKO liver may be responsible for the extensive increases of H3K27me3 in down-regulated and unchanged gene loci.

*Hnf4a* deficiency in young-adult mouse liver causes a global increase in H3K4ac. H3K4 acetylation can play a positive role in transcription [39] or mediate the termination of transcription to allow heterochromatin reassembly [43]. *Hnf4a* deficiency increases H3K4ac in most of the investigated gene loci regardless of up-, down-regulated, or unchanged genes, suggesting that H3K4ac may have a complex role in gene regulation. A previous study suggests that *HDAC6* is a target gene of HNF4α in humans [21]. However, *Hnf4a* deficiency does not affect hepatic expression of *Hdac6* or *Hdac3*. Nevertheless, the loss of HNF4α might perturb the recruitment of HDACs to HNF4α-target genes [19], contributing to the increase in H3K4ac.

The redundant combination of multiple active/suppressive histone modifications to ensure robust chromatin regulation and the existence of bivalent domains indicate the complex nature of gene regulation by histone modifications [13], [15]. *Hnf4a* deficiency increases both active signatures (e.g. H3K4ac) and repressive signatures (e.g. H3K9me2) at the same loci. The promoter of a gene locus with unchanged mRNA expression, such as *Ugt2b36*, has both increased active signature H3K4ac and increased repressive signatures H3K4me2, H3K9me2, and H3K9me3 due to *Hnf4a* deficiency, further confirming the complex nature of histone modifications in regulating gene expression. The final impact on gene expression may be determined by the combination of these active/suppressive marks, the dominant histone modification (if exists) and/or other factors such as the coactivators or corepressors that recognize these histone modifications. In the present study, *Hnf4a* deficiency increases the expression of histone H1 isoform H1.2 and Histone H3 variant H3.3. The histone linker H1 stimulates H3K27me3 by EZH2 [44] and has a major role in regulating global chromatin structure [45]. The histone H3 variant H3.3 is enriched at the transcription start sites of active and repressed genes and in the bodies of transcribed sequences [46]. H3.3 can be incorporated into chromatin in non-proliferating cells; thus, induction of H3.3 may allow adaptive regulation of chromatin structure and gene expression in the *Hnf4a*-deficient liver.

*Hnf4a* deficiency in the young-adult mouse liver causes a global increase in H3K4me3. The present data suggests a role of induced *Setd7* and *Kmt2c*, two H3K4 methyltransferases [32], [47], in the increase of total H3K4me2 and H3K4me3 responsive to *Hnf4a* deficiency. In contrast to other histone modifications investigated, H3K4me3 alterations correlate bidirectionally with gene expression, at least for the loci investigated, in that the increases in H3K4me3 correspond with increased gene expression, and decreases in H3K4me3 correspond with decreased gene expression. H3K4me3 interacts directly with the TAF3 subunit of the general initiation factor TFIID to facilitate the recruitment of TFIID, and H3K4me3 cooperates with the TATA Box to enhance the assembly of the preinitiation complex and initiation of transcription [48]. We hypothesize that H3K4me3 might be a dominant histone modification for gene expression in somatic cells, which needs to be further investigated. Additionally, the finding that *Hnf4a* deficiency affects H3K4me3 in exon1 of *Cyp2c44* as well as exon1 and exon2 of *Ugt2b1* but not in the corresponding promoters suggests that the alteration of H3K4me3 may be gene locus specific. Future genome-wide mapping will provide comprehensive information regarding gene locus-specific H3K4me3 alteration in response to *Hnf4a* deficiency as well as the role of H3K4me3 in hepatic gene expression regulated by HNF4α.

It is noteworthy that sex differences exist in the response to liver-specific *Hnf4a* deficiency, and HNF4α has gender-divergent expression, with 5 fold more protein in male than female rat livers [18]. Thus, the impact of *Hnf4a* deficiency on DNA and histone modifications in male mouse liver might be more dramatic, which warrants further investigation.

In conclusion, the present study provides convenient improved (h)MeDIP- and ChIP-qPCR assays for epigenetic study. *Hnf4a* deficiency in young-adult mouse hepatocytes alters H3K4me2, H3K4me3, H3K9me2, H3K9me3, H3K27me3, and H3K4ac, as well as DNA methylation and hydroxymethylation to differential extents, at least for the loci investigated. The underlying mechanism may be induction of epigenetic enzymes responsible for the addition/removal of the epigenetic signatures, and/or the loss of HNF4α *per se* as a key coordinator for epigenetic modifiers. In addition to its key role in the regulation of transcriptome, HNF4α has a major role in regulating the epigenome in hepatocytes.

## Supporting Information

File S1 Figure S1, Effects of Hnf4a deficiency on histone H3 lysine 4 dimethyl (H3K4me2) and histone H3 lysine 4 trimethyl (H3K4me3) in female mouse livers. Enrichment of H3K4me2 and H3K4me3 at specific loci relative to input in ChIPed DNA fragments in livers from female *Hnf4a*-LivKO and wild-type mice was normalized by the positive control. Insets, the percentage of total ChIPed DNA fragments relative to input for H3K4me2 and H3K4me3 in livers from female *Hnf4a*-LivKO and wild-type mice. Negative = negative control. Mean ± S.E., N = 3 biological replicates. *, p<0.05 versus wild-type. Figure S2, Effects of Hnf4a deficiency on histone H3 lysine 9 dimethyl (H3K9me2) and histone H3 lysine 9 trimethyl (H3K9me3) in female mouse livers. Enrichment of H3K9me2 and H3K9me3 at specific loci relative to input in ChIPed DNA fragments in livers from female *Hnf4a*-LivKO and wild-type mice was normalized by the positive control. Insets, the percentage of total ChIPed DNA fragments relative to input for H3K9me2 and H3K9me3 in livers from female *Hnf4a*-LivKO and wild-type mice. Negative = negative control. Mean ± S.E., N = 3 biological replicates. *, p<0.05 versus wild-type. Figure S3, Effects of Hnf4a deficiency on histone H3 lysine 27 trimethyl (H3K27me3) and histone H3 lysine 4 acetylation (H3K4ac) in female mouse livers. Enrichment of H3K27me3 and H3K4acat specific loci relative to input for ChIPed DNA fragments in livers from female *Hnf4a*-LivKO and wild-type mice was normalized by the positive control. Insets, the percentage of total ChIPed DNA fragments relative to input for H3K27me3 and H3K4ac in livers from female *Hnf4a*-LivKO and wild-type mice. Negative = negative control. Mean ± S.E., N = 3 biological replicates. *, p<0.05 versus wild-type. Table S1, The efficiency (E) and coefficient of variation (CV) for Cq values in real-time PCR reactions in the presence/absence of input DNA sample and positive control. Table S2, The list of functions of genes tested in histone modifications due to *Hnf4a* deficiency. Table S3, List of qPCR primers used for IPed DNA fragments and cDNA.
(PDF)
