# Supplementary material for: Alterations of Epigenetic Signatures in Hepatocyte Nuclear Factor 4α Deficient Mouse Liver Determined by Improved ChIP-qPCR and (h)MeDIP-qPCR Assays
Source: PLoS One. 2014 Jan 10;9(1):e84925. doi: 10.1371/journal.pone.0084925 (PMC3888413; doi:10.1371/journal.pone.0084925)
Supplement: File S1 — Figure S1, Effects of Hnf4a deficiency on histone H3 lysine 4 dimethyl (H3K4me2) and histone H3 lysine 4 trimethyl (H3K4me3) in female mouse livers. Enrichment of H3K4me2 and H3K4me3 at specific loci relative to input in ChIPed DNA fragments in livers from female Hnf4a-LivKO and wild-type mice was normalized by the positive control. Insets, the percentage of total ChIPed DNA fragments relative to input for H3K4me2 and H3K4me3 in livers from female Hnf4a-LivKO and wild-type mice. Negative = negative control. Mean ± S.E., N = 3 biological replicates. *, p<0.05 versus wild-type. Figure S2, Effects of Hnf4a deficiency on histone H3 lysine 9 dimethyl (H3K9me2) and histone H3 lysine 9 trimethyl (H3K9me3) in female mouse livers. Enrichment of H3K9me2 and H3K9me3 at specific loci relative to input in ChIPed DNA fragments in livers from female Hnf4a-LivKO and wild-type mice was normalized by the positive control. Insets, the percentage of total ChIPed DNA fragments relative to input for H3K9me2 and H3K9me3 in livers from female Hnf4a-LivKO and wild-type mice. Negative = negative control. Mean ± S.E., N = 3 biological replicates. *, p<0.05 versus wild-type. Figure S3, Effects of Hnf4a deficiency on histone H3 lysine 27 trimethyl (H3K27me3) and histone H3 lysine 4 acetylation (H3K4ac) in female mouse livers. Enrichment of H3K27me3 and H3K4acat specific loci relative to input for ChIPed DNA fragments in livers from female Hnf4a-LivKO and wild-type mice was normalized by the positive control. Insets, the percentage of total ChIPed DNA fragments relative to input for H3K27me3 and H3K4ac in livers from female Hnf4a-LivKO and wild-type mice. Negative = negative control. Mean ± S.E., N = 3 biological replicates. *, p<0.05 versus wild-type. Table S1, The efficiency (E) and coefficient of variation (CV) for Cq values in real-time PCR reactions in the presence/absence of input DNA sample and positive control. Table S2, The list of functions of genes tested in histone modifications due [file pone.0084925.s001.pdf]

Table S1. The efficiency (E) and coefficient of variation (CV) for Cq values in real-time PCR reactions in the presence/absence of input DNA sample and positive control.

**Positive control (fg)**												
	0			1			10			100		
Input[a] (dilution fold)	2.5	50	500	2.5	50	500	2.5	50	500	2.5	50	500
CV (n=5)	0.68	0.77	1.45	1	1.12	0.78	1.12	0.78	1.03	0.78	1.03	1.18
E	1.96			1.92			1.95			1.91		
**Input[a] (dilution fold)**												
	0			2.5			50			500		
Positive control (fg)	1	10	100	1	10	100	1	10	100	1	10	100
CV (n=5)	1.26	1.28	1.86	1.15	0.21	0.77	1.1	1.15	0.54	0.5	0.82	0.4
E	1.9			1.91			1.9			1.94		

[a]Primers for Ugt2b36 promoter were used to determine potential interferences between the positive control and target genes in RT-PCR.

Table S2. The list of functions of genes tested in histone modifications due to *Hnf4a* deficiency.

Abbreviation	Full name	Main function
Defb1	Defensin beta 1	Antimicrobial activity, tumor suppresser, cellular differentiation[2]
Gadd45β	Growth arrest and DNA damage-inducible protein 45β	Activity-induced regulation of gene expression and DNA demethylation[3,4]
Cyp2c44, Cyp3a11, Sult1b1, Sult1e1, Ugt2b1, Ugt2b36	Cytochrome P450 2c44 and 3a11 Sulfotransferase 1b1 and 1e1 UDP glucuronosyltransferase 2b1 and 2b36	Phase I and II drug processing enzymes[5,6]
Asgr1	Asialoglycoprotein receptor 1	Clearance of glycoprotein and IgA, removal of apoptotic cells and low density lipoprotein, disposal of cellular fibronectin[7]
Gas2	Growth arrest specific 2	Regulates microfilament and cell shape changes during apoptosis[8]
Pdzk1	PDZ domain-containing 1	An adaptor primarily acting in the formation of diverse molecular complexes with its PDZ domains[9]
Perp	P53 apoptosis effector related to PMP-22	The role in the epithelial integrity of a number of tissues[10]
Celsr1	Cadherin, EGF LAG seven-pass G-type receptor	An atypical proto-cadherin that is involved in the regulation of several biological processes[11]
Lifr	leukemia inhibitory factor receptor	A pleiotropic cytokine present in both soluble and matrix-bound forms[12]
Ppara	Peroxisome proliferator-activated receptor α	Mediates the biological and toxicological effects of peroxisome proliferators[13]

Table S3. List of qPCR primers used for IPed DNA fragments and cDNA.

Primers for IPed DNA fragments			
**Gene**	**Sequence[a]**	**Product (bp)**	**Tm**
*Rgl3*_intron 2-3	CAATTATACCCATCTCTGGCAC	136	60
	AGTGCTAGAGTAGTGGGGTGTC		
*Sox9*_exon3	CCTACAGCCCCTTCAACCTTCCTC	114	68
	CGGCTGCGTGACTGTAGTAGGAG		
*Zbp1*_exon1	CAGGTCCAAGCAGCCATTCTTGCC	134	68
	CTCAAGTCAACAGGAGCTTCTGCCAT		
*Casp9*_pro	TTTATGGAAGGAAATGTTGACTGA	111	60
	TGACCAGAACCAGAATAGACTC		
*Slc10a1*_pro	GTAAAGAATGAACCCTGTGCTG	159	60
	GCTGTGGGCAAATAAAAGATTATG		
*Slc10a1*_exon1	ATGGAGGCGCACAACGTATC	231	60
	GAAAGCACTGAGGGGCATGAT		
Slc47a1-exon1_2	ACATGGAACGCACGGAGGAGT	131	60
	ACCCGCCAGGACCAAGAGT		
Slc47a1-exon1	TGAGTCCAAGGTTGCAGATCC	149	60
	ACTCCTCCGTGCGTTCCATGT		
*Slc22a6*_exon1	CATCAGGACAAAGATTAAACGCT	246	60
	TGCCCTTTGCCTGAGGAGTA		
*Mgst3*_intron1-2	TTGTGCAGCAAACAGTTTACCT	173	60
	TGTGGAAGAAAGTACAACACAG		
*Slc15a2*_pro	GGTATTTTTCTCAAACAGATAACC	154	60
	AGCAGAACTTGAAGCTATAGGAA		
*Sult2a1*_Exon2	AGATTGTATGCTTGATTCAGACC	117	60
	CCTTATTGATTAATGCAGAATATCC		
*Gtf2h2*_pro	TTACGCTCTTGGGCACCACTT	139	60
	AAGTACCGGCAGTCTGAGTG		
*Gapdh*_pro	GACTCCTCGTCCTTAAGTTCAT	166	60
	TCTCTTTGGACCCGCCTCATT		
*Defb1*_pro	CACAGGACTTGGACTCTACTC	144	60
	GATGAGGCTTGAATGAGCTTGT		
*Gadd45b*_pro	ACATCCCTTCTTTCAGAGCTTG	161	60
	AGAAGTTTCTCTCCTGATTTCC		
*Sult1e1*_pro	GGTTAAAGGTAGAAAGGAGAAATG	173	60
	CATCTACACAGTAAATGCAATAGT		
*Asgr1*_pro	CAAGTATTGAACATGCGGAAGTG	152	60
	TAGACTTGGAACTGGGGAGGT		
*Cyp2C44*_pro	GGAGCGAGAACCAACATCCAA	133	60
	TTCCTTGTACTCAGAAATCCATAA		
*Cyp3a11*_pro	GCCTTGGTGAGAGGGTATTTG	153	60
	GCAGTTCGTATAGTTCATCCAC		

*Gas2*_pro	TCTTCTATTTGTAATGAACACTGG<br>GCACAATGTTAAAATTGGGTGTTG	114	60
*Pdzk1*_pro	GGCGGGGCTTGATTTTACTGA<br>GATGTATTTAACAGGGACGTGTG	118	60
*Perp*_pro	CTGAACCACCAGAGCACAGA<br>GCTTCCACAGGCAGATCCTA	100	60
*Sult1b1*_pro	CTTGTGTGCTGTTTTGTCTTGC<br>TCAACAGTTGTAATAGGTCAGAG	123	60
*Ugt2b1*_pro	CCATGCAACTCAAGAAGAAGG<br>CAAGGTTCACGAAGTTTGTCTTT	150	60
*Celsr1*_pro	CGCGGCCCCCGGTGTCCTC<br>CCGGCGCACCTCCGCATCCAC	124	69
*Lifr*_pro	CAAAGTGCAATGACAGCGGTTTGGGA<br>AGGGAGGTGCCTCTGCCGAGAC	171	68
*Ppara*_pro	CATGTGGACTCTGATCTTTGGA<br>GTATATAACAGTGAGCAAGAATGG	151	60
*Ugt2b36*_pro	CATGATTTTCCACCAACACAGTA<br>GTAATCCATCTGTCACTGCTTG	120	60
*Cyp2c44*_exon1	TTGCTCTCCTGGTTTTGGTGA<br>CAATAATGGGAAGTGGAGTGG	105	60
*Cyp2c44*_exon2	CGTGGTCCTACATGGCTATGA<br>CATGTCCCTTCTGGCTATCTT	110	60
*Sult1b1*_exon1	GCTTTGCTTAGATCCTTATTGAAC<br>CTTCAATCAGAACGAATGCTGC	121	60
*Sult1b1*_exon2	GAGTGCCTCAGAAGACGTTTG<br>GTAAGTGGTTATTACAATGTCACCT	136	60
*Sult1e1*_exon2	ATGAAGTTTTTGGAGAGTTCCGT<br>AGGATATGTAGCAATGACAAGGT	116	60
*Ugt2b1*_exon1	CCTACAGAATACAGCCATTGG<br>AATGAGGATGGAAGCAGAAGATA	105	60
*Ugt2b1*_exon2	TAACTGAGATGATGGGGAAGG<br>TGGCTGGTTTACAATGGAGTC	120	60

**Primers for cDNA**			
**Gene**	**Sequence**[a]	**Product (bp)**	**Tm**
Defb1	AGGTGTTGGCATTCTCACAAGT<br>TGGGCTTATCTGGTTTACAGG	134	60
Gadd45b	TTCACTTCACCCTGATCCAGT<br>ATTGCCTCTGCTCTCTTCACA	227	60
Sult1e1	TCCGTATGGTTCCTGGTATGA<br>GTTGAACGATTCTGTCCACAAG	176	60
Asgr1	GATCACATCCCAAAATTCCCAA<br>TTCCAGCTTCGACTCCACTAA	157	60
Cyp2c44	CCCACTCCACTTCCCATTATT<br>TTCCTTCACCACATCATAGCC	159	60

Cyp3a11	ACAAACAAGCAGGGATGGAC	84	60
	GGTAGAGGAGCACCAAGCTG		
Gas2	AACAAGCATGTGATGGTCCGA	193	60
	TAGCCTTGTAGGTGGCAGAGA		
Slc47a1	GAATTCCGCTGTCTCTCACGA	237	60
	ACTAAAATCCCACCCACCAAG		
Slc10a1	CTTCCAGCAAACCTTCAACAG	169	60
	ACCTTGAGTCTCTGAGCATTG		
Pdzk1	CACGTCACTCTGTTGGTCTGT	174	60
	TGTGCTGAGAGTTCGGTCTTT		
Perp	GCCATATTCCAGATCATCTCC	160	60
	AGCAGAAGAAGAAGGAAC		
Sult1b1	CCCTAAATCAGGTACTACTTG	229	60
	GTTCTCCCAGAAGGATTTTGG		
Ugt2b1	GTTTGATGTCGTTCTAGCAGAT	166	60
	TGACAGAACCACAGGCACATA		
Celsr1	GCTGCTGTCCCTCTTTGTAG	119	60
	AAGTCACGTTCAGGATGTTGG		
Lifr	TGTGGTGACCAAGGAAAACTC	121	60
	CTTAATCCATTCTCGCTTCCG		
Ppara	ATGAAGAGGGCTGAGCGTAG	194	60
	AAACGCAACGTAGAGTGCTGT		
Reg1	GCCAGGAAGCTGAAGAAGACC	172	60
	GCCTGACTGAGAACTGACACC		
Ugt2b36	TGTCATTTTGTCAGATGCTATTG	120	60
	CCTCCACTGTATTTTTCAAGATA		
Gapdh	CAGCAAGGACACTGAGCAAGA	85	60
	GGGTCTGGGATGGAAATTGTG		
Setd7	GAATTACACACCAAGAGGTTGAC	171	60
	CATAGACGCAGTTCGGAGTGA		
Kmt2c	GCCAGGCATTTATTGAGTTGA	170	60
	AGGCTGTCTTATGGGGTCTGT		
Wdr5	CAGTCTCAGCCGTTCATTTCA	153	60
	GAGAGAACTTCACGAAGGACA		
Ehmt2	CAAGGATGGCGAGGTTTACTG	153	60
	CTGGAGCTGAAGAAGGCAATG		
Suv39h1	GCCACGGCAGAATCTAAAATG	229	60
	TACCTCATTCTCCACGGTGAT		
Ezh2	TTACTGCTGGCACCGTCTGA	195	60
	GGTTGCATCCACCACAAAATCA		
Hdac3	GATGGCATTGATGACCAGAGT	156	60
	ATGTCCTCGAATGCTGAGATTG		
Hdac6	AGAGTAGGCACAATCCCCAGT	166	60
	CCCACAACTAGGTCCTCTTCA		

Dnmt1	GGACAGTGACACCCTTTTTGA<br>TCTTCCAGTTTCTCCACAGCA	234	60
Tet2	TCCCAACACGGAACTACAATG<br>GAATGGGCAGAAACTGTAGCA	133	60
Tet3	TTACACTCACCTGGGATCTGG<br>AGTGTGTGTCTTCGGATCACC	174	60
Idh1	AGATGCAAGAAGATGAAATGACA<br>TGACGCCCACGTTGTATTTCT	180	60
Idh2	ATGGGAACCAGGACCTTATCA<br>TCCAGGTTGCTCTTAATGGTG	181	60
Idh3a	CCACAACACAAAACAGGTGAC<br>CTTTCCTCCTGGTCCTTGAAT	181	60
Hist1h1c	GTTCGTATTGGACTGCAAGGT<br>CTCCCAAACTTCCAGGCTAAC	197	60
Hist1h1d	CAAGAAGGTGAAGGCTGCTAA<br>AAAGCAGGACGCACCACTCTA	160	60
H3F3b	AGAGATCCGTCGTTACCAGAA<br>ATCTTCAAACAACCCCACCAG	172	60

[a]Up: Forward; Down: Reverse

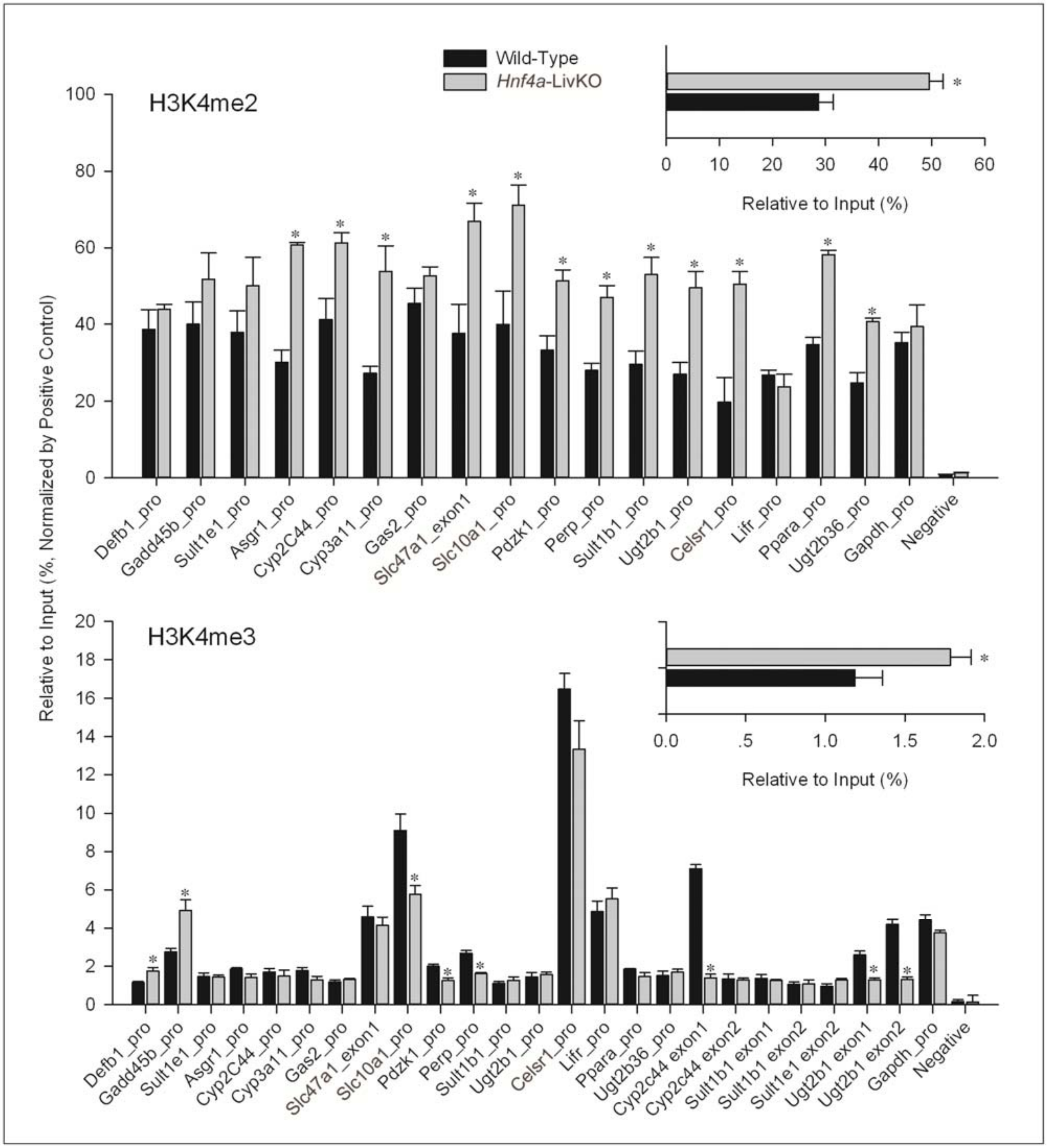

Wild-Type
Hnf4a-LivKO
H3K4me2
H3K4me3
Relative to Input (%, Normalized by Positive Control)
Relative to Input (%)
Relative to Input (%)
Defb1_pro
Gadd45b_pro
Sult1e1_pro
Asgr1_pro
Cyp2C44_pro
Cyp3a11_pro
Gas2_pro
Slc47a1_exon1
Slc10a1_pro
Pdzk1_pro
Perp_pro
Sult1b1_pro
Ugt2b1_pro
Celsr1_pro
Lifr_pro
Ppara_pro
Ugt2b36_pro
Gapdh_pro
Negative
Cyp2c44 exon1
Cyp2c44 exon2
Sult1b1 exon1
Sult1b1 exon2
Sult1e1 exon2
Ugt2b1 exon1
Ugt2b1 exon2

Fig. S1.

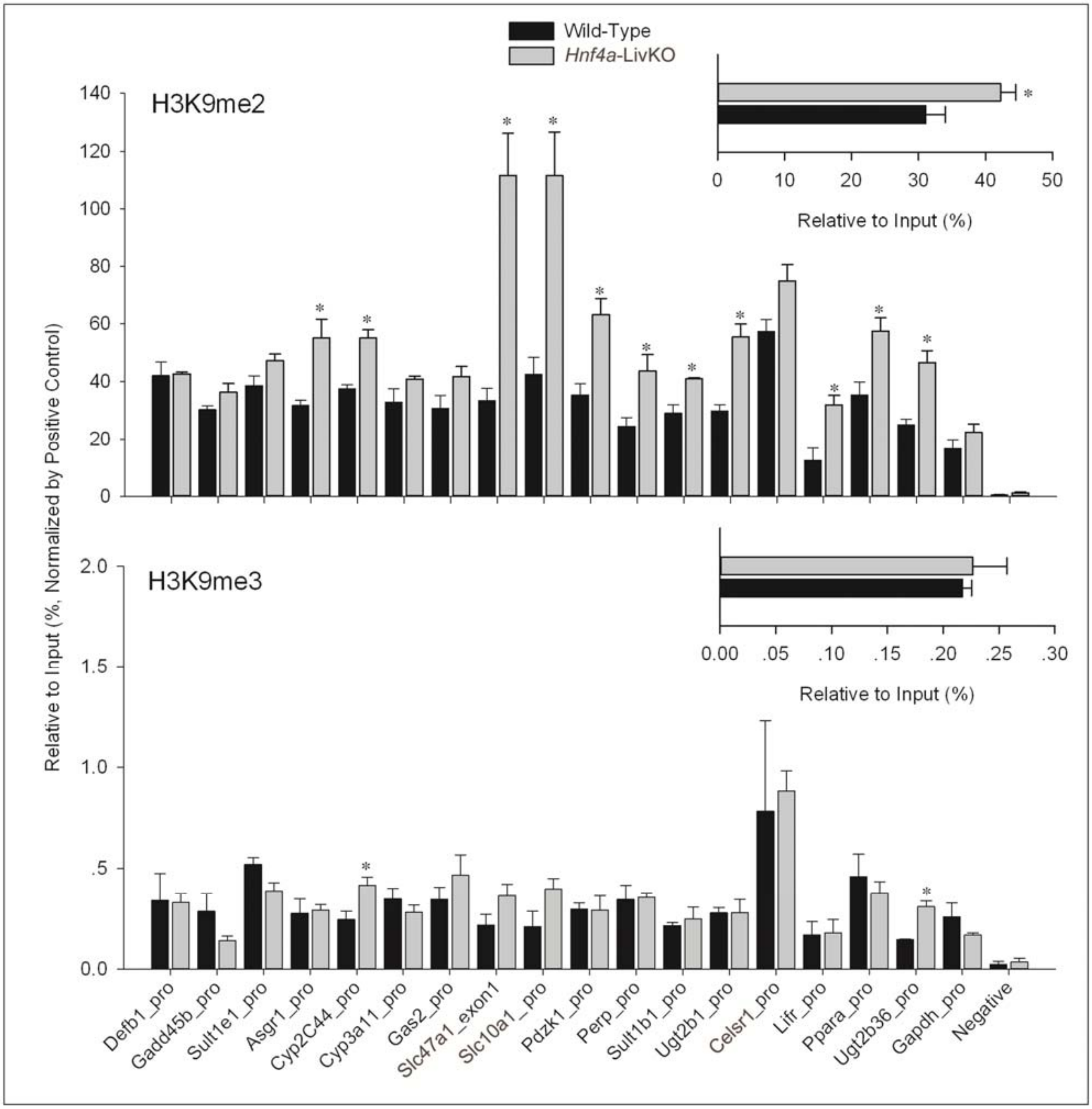
Wild-Type
Hnf4a-LivKO
H3K9me2
H3K9me3
Relative to Input (%, Normalized by Positive Control)
Relative to Input (%)
Relative to Input (%)
Defb1_pro
Gadd45b_pro
Sult1e1_pro
Asgr1_pro
Cyp2C44_pro
Cyp3a11_pro
Gas2_pro
Slc47a1_exon1
Slc10a1_pro
Pdzk1_pro
Perp_pro
Sult1b1_pro
Ugt2b1_pro
Celsr1_pro
Lifr_pro
Ppara_pro
Ugt2b36_pro
Gapdh_pro
Negative

Fig. S2.

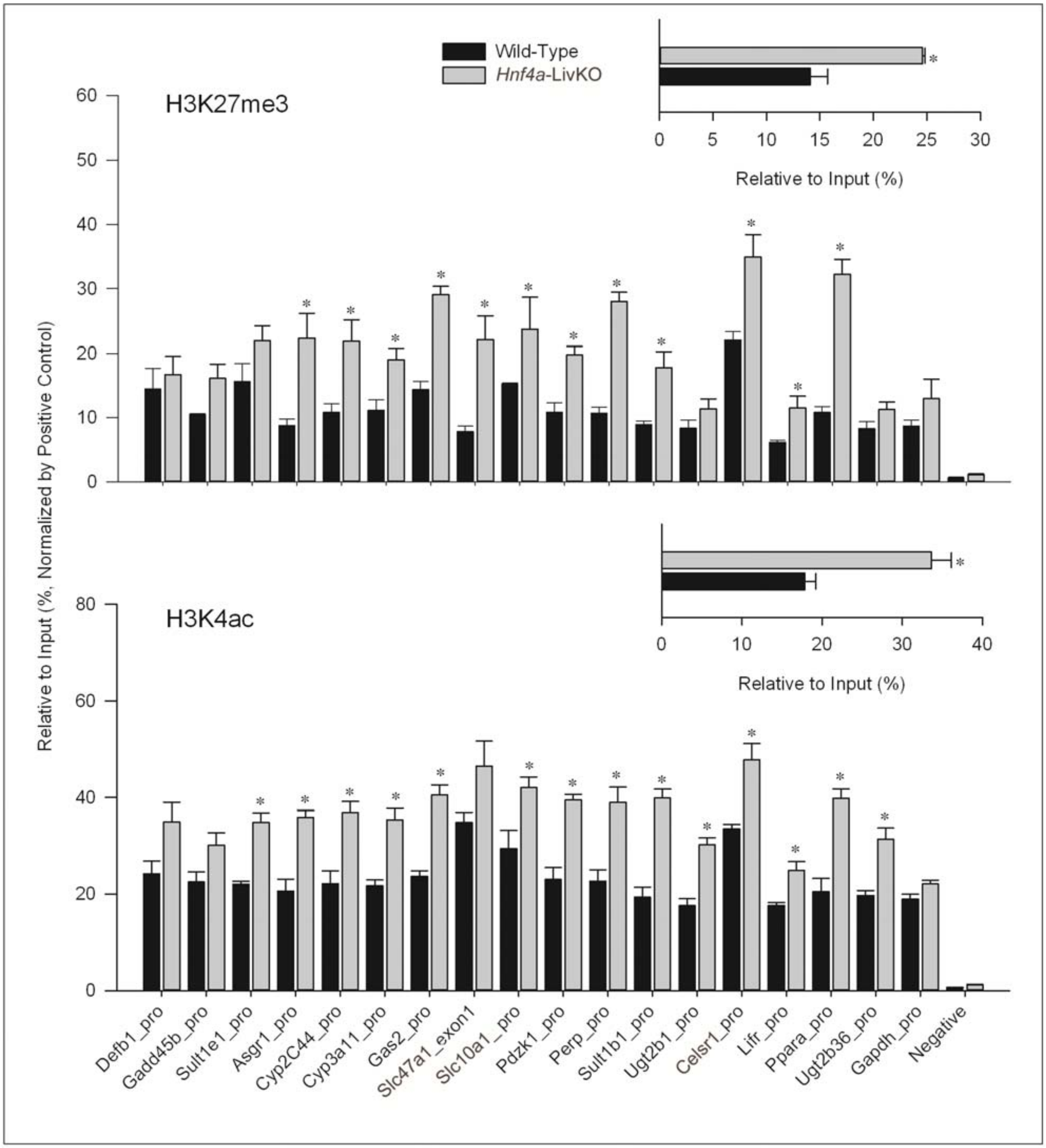

Wild-Type
Hnf4a-LivKO
H3K27me3
H3K4ac
Relative to Input (%)
Relative to Input (%, Normalized by Positive Control)
60
50
40
30
20
10
0
80
0
5
15
25
30
Defb1_pro
Gadd45b_pro
Sult1e1_pro
Asgr1_pro
Cyp2C44_pro
Cyp3a11_pro
Gas2_pro
Slc47a1_exon1
Slc10a1_pro
Pdzk1_pro
Perp_pro
Sult1b1_pro
Ugt2b1_pro
Celsr1_pro
Lifr_pro
Ppara_pro
Ugt2b36_pro
Gapdh_pro
Negative

Fig. S3.

References

1. Lu, H., Gunewardena, S., Cui, J. Y., Yoo, B., Zhong, X. B. & Klaassen, C. D. (2013). RNA-sequencing quantification of hepatic ontogeny and tissue distribution of mRNAs of phase II enzymes in mice. *Drug Metab Dispos.* **41**, 844-57.
2. Prado-Montes de Oca, E. (2010). Human beta-defensin 1: a restless warrior against allergies, infections and cancer. *Int J Biochem Cell Biol.* **42**, 800-4.
3. Chen, Z., Clark, S., Birkeland, M., Sung, C. M., Lago, A., Liu, R., Kirkpatrick, R., Johanson, K., Winkler, J. D. & Hu, E. (2002). Induction and superinduction of growth arrest and DNA damage gene 45 (GADD45) alpha and beta messenger RNAs by histone deacetylase inhibitors trichostatin A (TSA) and butyrate in SW620 human colon carcinoma cells. *Cancer Lett.* **188**, 127-40.
4. Gavin, D. P., Sharma, R. P., Chase, K. A., Matrisciano, F., Dong, E. & Guidotti, A. (2012). Growth arrest and DNA-damage-inducible, beta (GADD45b)-mediated DNA demethylation in major psychosis. *Neuropsychopharmacology.* **37**, 531-42.
5. Lu, H., Gonzalez, F. J. & Klaassen, C. (2010). Alterations in hepatic mRNA expression of phase II enzymes and xenobiotic transporters after targeted disruption of hepatocyte nuclear factor 4 alpha. *Toxicol Sci.* **118**, 380-90.
6. Elshenawy, O. H., Anwar-Mohamed, A., Abdelhamid, G. & El-Kadi, A. O. (2012). Murine atrial HL-1 cell line is a reliable model to study drug metabolizing enzymes in the heart. *Vascul Pharmacol*. **58**, 326-33
7. Rigopoulou, E. I., Roggenbuck, D., Smyk, D. S., Liaskos, C., Mytilinaiou, M. G., Feist, E., Conrad, K. & Bogdanos, D. P. (2012). Asialoglycoprotein receptor (ASGPR) as target autoantigen in liver autoimmunity: lost and found. *Autoimmun Rev.* **12**, 260-9.
8. Benetti, R., Del Sal, G., Monte, M., Paroni, G., Brancolini, C. & Schneider, C. (2001). The death substrate Gas2 binds m-calpain and increases susceptibility to p53-dependent apoptosis. *EMBO J.* **20**, 2702-14.
9. Kim, J. K., Kwon, O., Kim, J., Kim, E. K., Park, H. K., Lee, J. E., Kim, K. L., Choi, J. W., Lim, S., Seok, H., Lee-Kwon, W., Choi, J. H., Kang, B. H., Kim, S., Ryu, S. H. & Suh, P. G. (2012). PDZ domain-containing 1 (PDZK1) protein regulates phospholipase C-beta3 (PLC-beta3)-specific activation of somatostatin by forming a ternary complex with PLC-beta3 and somatostatin receptors. *J Biol Chem.* **287**, 21012-24.
10. Ihrie, R. A., Marques, M. R., Nguyen, B. T., Horner, J. S., Papazoglu, C., Bronson, R. T., Mills, A. A. & Attardi, L. D. (2005). Perp is a p63-regulated gene essential for epithelial integrity. *Cell.*

120, 843-56.

11. Carreira-Barbosa, F., Kajita, M., Morel, V., Wada, H., Okamoto, H., Martinez Arias, A., Fujita, Y., Wilson, S. W. & Tada, M. (2009). Flamingo regulates epiboly and convergence/extension movements through cell cohesive and signalling functions during zebrafish gastrulation. *Development* .**136**, 383-92.

12. Nogueira-Silva, C., Piairo, P., Carvalho-Dias, E., Peixoto, F. O., Moura, R. S. & Correia-Pinto, J. (2012). Leukemia inhibitory factor in rat fetal lung development: expression and functional studies. *PLoS One.* **7**, e30517.

13. Aoyama, T., Peters, J. M., Iritani, N., Nakajima, T., Furihata, K., Hashimoto, T. & Gonzalez, F. J. (1998). Altered constitutive expression of fatty acid-metabolizing enzymes in mice lacking the peroxisome proliferator-activated receptor alpha (PPARalpha). *J Biol Chem.* **273**, 5678-84.
